# Global, regional, and national burden of bone fractures in 204 countries and territories, 1990–2019: a systematic analysis from the Global Burden of Disease Study 2019

**DOI:** 10.1016/S2666-7568(21)00172-0

**Published:** 2021-09

**Authors:** GBD 2019 Fracture Collaborators, Ai-Min Wu, Catherine Bisignano, Spencer L James, Gdiom Gebreheat Abady, Aidin Abedi, Eman Abu-Gharbieh, Robert Kaba Alhassan, Vahid Alipour, Jalal Arabloo, Malke Asaad, Wondwossen Niguse Asmare, Atalel Fentahun Awedew, Maciej Banach, Srikanta K Banerjee, Ali Bijani, Tesega Tesega Mengistu Birhanu, Srinivasa Rao Bolla, Luis Alberto Cámera, Jung-Chen Chang, Daniel Youngwhan Cho, Michael T Chung, Rosa A S Couto, Xiaochen Dai, Lalit Dandona, Rakhi Dandona, Farshad Farzadfar, Irina Filip, Florian Fischer, Artem Alekseevich Fomenkov, Tiffany K Gill, Bhawna Gupta, Juanita A Haagsma, Arvin Haj-Mirzaian, Samer Hamidi, Simon I Hay, Irena M Ilic, Milena D Ilic, Rebecca Q Ivers, Mikk Jürisson, Rohollah Kalhor, Tanuj Kanchan, Taras Kavetskyy, Rovshan Khalilov, Ejaz Ahmad Khan, Maseer Khan, Cameron J Kneib, Vijay Krishnamoorthy, G Anil Kumar, Narinder Kumar, Ratilal Lalloo, Savita Lasrado, Stephen S Lim, Zichen Liu, Ali Manafi, Navid Manafi, Ritesh G Menezes, Tuomo J Meretoja, Bartosz Miazgowski, Ted R Miller, Yousef Mohammad, Abdollah Mohammadian-Hafshejani, Ali H Mokdad, Christopher J L Murray, Mehdi Naderi, Mukhammad David Naimzada, Vinod C Nayak, Cuong Tat Nguyen, Rajan Nikbakhsh, Andrew T Olagunju, Nikita Otstavnov, Stanislav S Otstavnov, Jagadish Rao Padubidri, Jeevan Pereira, Hai Quang Pham, Marina Pinheiro, Suzanne Polinder, Hadis Pourchamani, Navid Rabiee, Amir Radfar, Mohammad Hifz Ur Rahman, David Laith Rawaf, Salman Rawaf, Mohammad Reza Saeb, Abdallah M Samy, Lidia Sanchez Riera, David C Schwebel, Saeed Shahabi, Masood Ali Shaikh, Amin Soheili, Rafael Tabarés-Seisdedos, Marcos Roberto Tovani-Palone, Bach Xuan Tran, Ravensara S Travillian, Pascual R Valdez, Tommi Juhani Vasankari, Diana Zuleika Velazquez, Narayanaswamy Venketasubramanian, Giang Thu Vu, Zhi-Jiang Zhang, Theo Vos

## Abstract

**Background:**

Bone fractures are a global public health issue; however, to date, no comprehensive study of their incidence and burden has been done. We aimed to measure the global, regional, and national incidence, prevalence, and years lived with disability (YLDs) of fractures from 1990 to 2019.

**Methods:**

Using the framework of the Global Burden of Diseases, Injuries, and Risk Factors Study (GBD) 2019, we compared numbers and age-standardised rates of global incidence, prevalence, and YLDs of fractures across the 21 GBD regions and 204 countries and territories, by age, sex, and year, from 1990 to 2019. We report estimates with 95% uncertainty intervals (UIs).

**Findings:**

Globally, in 2019, there were 178 million (95% UI 162–196) new fractures (an increase of 33·4% [30·1–37·0] since 1990), 455 million (428–484) prevalent cases of acute or long-term symptoms of a fracture (an increase of 70·1% [67·5–72·5] since 1990), and 25·8 million (17·8–35·8) YLDs (an increase of 65·3% [62·4–68·0] since 1990). The age-standardised rates of fractures in 2019 were 2296·2 incident cases (2091·1–2529·5) per 100 000 population (a decrease of 9·6% [8·1–11·1] since 1990), 5614·3 prevalent cases (5286·1–5977·5) per 100 000 population (a decrease of 6·7% [5·7–7·6] since 1990), and 319·0 YLDs (220·1–442·5) per 100 000 population (a decrease of 8·4% [7·2–9·5] since 1990). Lower leg fractures of the patella, tibia or fibula, or ankle were the most common and burdensome fracture in 2019, with an age-standardised incidence rate of 419·9 cases (345·8–512·0) per 100 000 population and an age-standardised rate of YLDs of 190·4 (125·0–276·9) per 100 000 population. In 2019, age-specific rates of fracture incidence were highest in the oldest age groups, with, for instance, 15 381·5 incident cases (11 245·3–20 651·9) per 100 000 population in those aged 95 years and older.

**Interpretation:**

The global age-standardised rates of incidence, prevalence, and YLDs for fractures decreased slightly from 1990 to 2019, but the absolute counts increased substantially. Older people have a particularly high risk of fractures, and more widespread injury-prevention efforts and access to screening and treatment of osteoporosis for older individuals should help to reduce the overall burden.

**Funding:**

Bill & Melinda Gates Foundation.

## Introduction

Bone fractures are a public health issue around the world and pose a serious economic burden,^1^, ^2^ especially in people with osteoporosis.^3^ Fractures can lead to work absence, decreased productivity, disability, impaired quality of life, health loss, and high health-care costs and are a major burden to individuals, families, societies, and health-care systems.^4^, ^5^, ^6^ From a meta-analysis of 113 studies, the pooled cost for treatment in hospital for a hip fracture was estimated to be US$10 075, and total health and social care costs for one hip fracture after 12 months amounted to a global mean of $43 669.^7^

Previous epidemiological studies of fractures have focused on specific regions or countries^8^, ^9^, ^10^, ^11^ or specific types of fractures or anatomical sites.^12^, ^13^ Furthermore, these studies did not measure the relative disability caused by fractures at different anatomical sites.

The Global Burden of Diseases, Injuries, and Risk Factors Study (GBD) is the product of a global research collaboration to quantify the worldwide effects of hundreds of diseases, injuries, and risk factors. Using the updated framework of GBD 2019,^14^ we describe the incidence, prevalence, and years lived with disability (YLDs) for fractures (including total fractures and fracture subcategories). This is the first effort to quantify the burden of fractures at global, regional, and national levels for all ages, both sexes, and over time from 1990 to 2019. The results of this study will help to identify the current burden of fractures and facilitate development of global, regional, and national responses to support the prevention and treatment of fractures. This manuscript was produced as part of the GBD Collaborator Network and in accordance with the GBD Protocol.

**Research in context**

**Evidence before this study**

To our knowledge, the Global Burden of Diseases, Injuries, and Risk Factors Study (GBD) 2019 was the first systematic assessment to report the burden of bone fractures at all fracture sites at the global, regional, and national level. Before GBD 2019, fractures were part of the GBD analytical process to calculate disability, but estimates were not available as separate results. For GBD 2019, fracture burden results were available in online tools, but no article had been published on the burden of all anatomical fracture sites. Outside of GBD, fracture-related studies have focused only on some anatomical sites or types of fractures, or the burden of fractures in a particular location or locations.

**Added value of this study**

This study is the most comprehensive analysis of the burden of fractures to date, providing estimates of incidence, prevalence, and years lived with disability for fractures at 12 anatomical sites, globally and for 21 GBD regions and 204 countries and territories, and among all age groups and both sexes, from 1990 to 2019. Furthermore, we provide estimates for fracture burden by the injury that caused the fracture, such as a fall. This study expands our understanding of the burden of fractures, particularly for locations and at anatomical sites not previously assessed.

**Implications of all the available evidence**

The findings from this study can help guide evidence-based fracture prevention, mitigation, treatment, and resource allocation efforts. This study will allow policy makers to prioritise locations and age groups with the highest incidence and disability due to fractures. Furthermore, it provides crucial information for policy makers and medical professionals on the most burdensome fracture sites and the types of injury that contribute to the largest burdens from fractures.

## Methods

### Overview

Our approach to estimating the burden of fractures was developed within the GBD 2019 framework. GBD 2019 provides a comprehensive assessment by age and sex of all-cause and cause-specific incidence, prevalence, mortality, and disability for 369 diseases and injuries and 87 risk factors in 204 countries and territories from 1990 to 2019 using standardised analytical methods.^14^, ^15^ Country-level data are further aggregated into 21 GBD regions; information on these regions has been published previously.^16^ There are also 23 GBD age categories, from age 0–6 days to 95 years and older. GBD 2019 aims to use all available disease and injury data from a range of data source types including civil registration and vital statistics, household surveys, and hospital records.^14^ Within the GBD 2019 framework, fractures are considered a nature of injury that occurs as a function of the cause of injury. For example, a fracture of the vertebral column is the nature of injury that might result from interpersonal violence, and a fracture of the hip is the nature of injury that might result from a cyclist road injury

### Causes of injury

GBD classifies causes into a hierarchy with four levels: Level 1 causes are broad aggregate categories (injuries; non-communicable diseases; and communicable, maternal, neonatal, and nutritional diseases), with increasing specificity up to Level 4 causes, which are the most specific (eg, motor vehicle road injury).^14^ Details on injuries data sources have been published previously.^17^ Most data sources on causes of injury (such as road injury or fall) came from hospital inpatient records complemented by some survey, injury surveillance, and emergency department data. The incidence of 29 external causes of injury was modelled in disease model—Bayesian meta-regression version 2.1 (DisMod-MR 2.1), a tool that estimates a consistent set of incidence, prevalence, and mortality estimates from sparse and heterogeneous epidemiological data and produces estimates for every cause, age group, sex, year (from 1990 to 2019), and geographical location. We used data from the US National Hospital Ambulatory Medical Care Survey to estimate the ratio of inpatient to outpatient incidence using a Bayesian meta-regression tool, MR-BRT, with a spline on age. We applied these ratios to all outpatient data before modelling incidence rates in DisMod-MR 2.1. Subsequently, we used the same ratios to extrapolate estimates of outpatient injury incidence from the modelled hospitalised incidence. Incidence data presented throughout are the incidence of hospitalised plus outpatient injury. A more detailed description of injury estimation has been published previously.^14^

### Nature of injury

We used clinical record data from 28 countries coded for both cause and nature of the injury to estimate the proportion of each cause that resulted in each nature of injury^14^—eg, the proportion of falls that resulted in fracture of the pelvis compared with fracture of the skull. In this study, we focus on bone fractures. We included 12 fracture sites: skull; facial bones; sternum or one or more ribs, or both; clavicle, scapula, or humerus; radius or ulna, or both; hand, wrist, and other distal part of hand; vertebral column; pelvis; hip; femur, other than femoral neck; patella, tibia or fibula, or ankle; and foot bones except ankle.

If an injury resulted in fractures in multiple anatomical sites, we chose the most severe fracture on the basis of their disability weights. GBD disability weights were derived from household surveys in nine countries (Bangladesh, Hungary, Indonesia, Italy, the Netherlands, Peru, Sweden, Tanzania, and the USA) and a web-based survey published in English, Spanish, and Mandarin.^14^ The weights represent the relative severity of one health state compared with all other health states.^18^ More on short-term and long-term disability weights by fracture site is in the appendix (p 21). For each cause of injury, we estimated the proportions resulting in the 12 fracture categories and 33 other nature of injury categories using a multinomial regression that ensures proportions of all nature of injury categories for a single cause of injury sum to 1. This process produced incidence estimates for each cause and nature of injury combination for each location, age group, sex, and year.

### Prevalence and YLDs

We calculated prevalence for each acute injury on the basis of the average time until recovery from Dutch Injury Surveillance System data.^19^ For untreated cases of injuries, the duration of injury was based on consensus expert opinion. We assumed that countries with a Healthcare Access and Quality (HAQ) Index score of 75 or higher (on a scale of 0–100) would provide universal access to injury treatments and assumed a linear decrease in access to 10% at an HAQ Index score of zero.^20^ For each fracture site, we estimated a probability of long-term disability from seven studies^21^, ^22^, ^23^, ^24^, ^25^, ^26^, ^27^ with general health status measured with the 12-Item Short Form Survey (SF-12) or EQ-5D. Long-term disability is defined as having lower functional status 1 year after fracture than just before the fracture. Six of these studies^21^, ^22^, ^23^, ^24^, ^25^, ^26^ followed up patients for at least 12 months after an injury. The seventh source was the Medical Expenditure Panel Survey (MEPS)^27^ in the USA, in which each individual was asked to complete the SF-12 twice over a 2-year period, allowing comparison of health status in those with a measurement before and after a reported injury. To translate SF-12 scores into the space between 0 and 1 of the GBD disability weights, we surveyed staff of the Institute for Health Metrics and Evaluation and participants of annual GBD training workshops using convenience sampling strategies. Respondents were asked to complete SF-12 questionnaires based on 60 health states with lay descriptions of the main symptoms and functional limitations that were also used in the GBD disability weight surveys.^18^ We calculated the amount of disability using a linear regression on the logit of disability weight value for each individual from their SF-12 score, with dummies for all nature of injury categories, using as reference the no-injury cases from MEPS respondents before their injury. The amount of disability as measured at 1 year minus the estimated pre-injury disability was divided by the disability weight for each long-term injury outcome as an estimate of the probability of long-term disability for each fracture. These methods have been published previously.^14^, ^17^ We then converted cause-nature incidence rates of long-term disability to prevalence using the differential equation solver built into DisMod-MR 2.1. We incorporated excess mortality for those with long-term consequences after a hip fracture from a meta-analysis of studies of standardised mortality ratios.^28^, ^29^ This process produced prevalence estimates for each cause-nature combination for each fracture anatomical site, including all fractures combined. Detailed descriptions of these methods have been published previously.^17^

We calculated YLDs by multiplying prevalence by the disability weight for each anatomical site. YLDs were adjusted for comorbidity with any of the 369 diseases and injuries included in GBD 2019 in a micro-simulation.

### Statistical analysis

We present estimates in counts and age-standardised rates per 100 000 population using the GBD standard population structure.^30^ Consistent with the GBD framework, we provide 95% uncertainty intervals (UIs) for all estimates. We calculated final estimates using the mean estimate across 1000 draws, with the 25th and 975th ranked values across all 1000 draws as the lower and upper bounds of the 95% UIs. As part of GBD 2019, this study follows the Guidelines for Accurate and Transparent Health Estimates Reporting (GATHER) recommendations.^14^, ^31^

### Role of the funding source

The funder of the study had no role in study design, data collection, data analysis, data interpretation, or the writing of the report.

## Results

In 2019, the global number of new cases of fracture was estimated to be 178 million (95% UI 162–196), an increase of 33·4% (30·1–37·0) since 1990 (table 1; appendix pp 2–9). The number of new cases of fractures was 102 million (93·4–111) in males compared with 76·4 million (68·7–85·3) in females. The age-standardised incidence rate of fracture was 2296·2 cases (2091·1–2529·5) per 100 000 population in 2019, a 9·6% (8·1–11·1) decrease since 1990 (table 2). The age-standardised rate in males was 2619·8 cases (2406·1–2865·7) per 100 000 population, which is higher than that in females (1943·6 cases [1739·7–2176·2] per 100 000 population).

**Table 1:** Global number of incident cases, prevalent cases, and years lived with disability of fractures by anatomical site and sex in 2019, and percentage change from 1990 to 2019

	**Incidence (millions)**		**Prevalence (millions)**		**Years lived with disability (millions)**	
	2019	Percentage change, 1990–2019 (%)	2019	Percentage change, 1990–2019 (%)	2019	Percentage change, 1990–2019 (%)
**Fractures**						
Females	76·4 (68·7–85·3)	43·3% (39·0–47·8)	200 (188–213)	76·7% (74·1–79·6)	11·5 (7·96–15·8)	71·3% (68·3–74·4)
Males	102 (93·4–111)	26·8% (23·8–30·1)	255 (240–272)	65·2% (62·7–67·5)	14·4 (9·84–20·1)	60·8% (58·0–63·4)
Both	178 (162–196)	33·4% (30·1–37·0)	455 (428–484)	70·1% (67·5–72·5)	25·8 (17·8–35·8)	65·3% (62·4–68·0)
**Fracture of skull**						
Females	2·44 (1·82–3·37)	20·9% (6·3–29·9)	1·26 (1·13–1·48)	76·2% (66·8–84·3)	0·0833 (0·0570–0·120)	72·8% (62·7–81·8)
Males	5·16 (4·08–6·56)	16·4% (6·9–23·8)	1·92 (1·71–2·19)	59·2% (51·8–66·3)	0·130 (0·0873–0·186)	57·0% (49·1–65·4)
Both	7·59 (5·91–9·87)	17·8% (7·1–25·7)	3·18 (2·85–3·66)	65·5% (57·6–73·1)	0·213 (0·144–0·308)	62·8% (54·7–71·1)
**Fracture of facial bones**						
Females	3·53 (2·66–4·71)	23·4% (11·3–32·2)	0·767 (0·648–0·925)	49·4% (40·0–57·2)	0·0491 (0·0302–0·0720)	47·2% (37·2–55·3)
Males	7·14 (5·81–8·87)	17·5% (9·4–23·7)	1·36 (1·16–1·60)	37·9% (31·0–44·1)	0·0885 (0·0542–0·130)	36·6% (29·5–43·2)
Both	10·7 (8·50–13·5)	19·4% (10·3–26·3)	2·13 (1·82–2·51)	41·8% (33·9–48·3)	0·138 (0·0843–0·201)	40·2% (32·3–47·1)
**Fracture of sternum or fracture of one or more ribs, or both**						
Females	1·50 (1·02–2·26)	52·5% (41·7–64·5)	0·741 (0·657–0·851)	72·3% (67·4–76·9)	0·0709 (0·0474–0·101)	70·3% (64·6–75·6)
Males	2·61 (1·91–3·59)	39·1% (32·1–46·8)	1·24 (1·11–1·40)	59·6% (55·4–63·5)	0·120 (0·0809–0·171)	58·0% (53·5–63·0)
Both	4·11 (2·97–5·82)	43·7% (35·7–52·8)	1·98 (1·77–2·26)	64·1% (59·8–67·9)	0·191 (0·128–0·272)	62·4% (57·8–66·9)
**Fracture of clavicle, scapula, or humerus**						
Females	8·50 (6·53–10·9)	38·4% (28·2–46·2)	3·45 (3·04–3·96)	69·7% (62·7–76·3)	0·114 (0·0692–0·179)	67·4% (60·3–74·3)
Males	10·8 (8·78–13·2)	24·6% (15·2–31·4)	3·95 (3·47–4·50)	53·6% (46·4–60·2)	0·133 (0·0818–0·207)	52·0% (44·6–59·0)
Both	19·3 (15·3–24·0)	30·3% (20·9–37·2)	7·40 (6·50–8·46)	60·7% (53·8–67·2)	0·247 (0·151–0·386)	58·8% (51·8–65·6)
**Fracture of radius or ulna, or both**						
Females	15·0 (11·5–19·5)	37·7% (30·5–43·6)	3·08 (2·55–3·79)	57·2% (51·2–63·3)	0·103 (0·0631–0·159)	60·6% (54·4–67·8)
Males	15·7 (13·0–19·0)	23·4% (17·1–28·2)	3·19 (2·69–3·77)	42·4% (36·8–47·8)	0·107 (0·0665–0·166)	46·4% (40·3–53·0)
Both	30·7 (24·5–38·6)	30·0% (23·4–34·9)	6·27 (5·25–7·55)	49·3% (43·6–54·7)	0·210 (0·131–0·325)	53·0% (47·2–59·4)
**Fracture of hand, wrist, or other distal part of hand**						
Females	6·71 (5·11–8·61)	28·1% (20·4–34·7)	9·51 (8·79–10·4)	76·5% (72·4–80·7)	0·120 (0·0633–0·208)	76·2% (72·2–80·7)
Males	12·3 (10·0–14·9)	16·6% (11·5–21·0)	14·2 (13·2–15·5)	60·8% (57·2–64·8)	0·181 (0·0962–0·314)	61·3% (57·8–65·9)
Both	19·0 (15·2–23·5)	20·4% (14·8–25·5)	23·7 (22·0–25·8)	66·7% (63·1–70·5)	0·301 (0·160–0·522)	66·9% (63·3–71·2)
**Fracture of vertebral column**						
Females	3·68 (2·71–5·04)	47·9% (29·4–60·2)	2·53 (2·19–2·97)	90·7% (78·3–100·9)	0·257 (0·173–0·361)	86·8% (74·5–97·1)
Males	4·90 (3·87–6·28)	30·9% (18·5–40·2)	2·80 (2·39–3·25)	69·0% (59·1–78·2)	0·292 (0·194–0·409)	66·2% (56·5–75·5)
Both	8·58 (6·64–11·3)	37·7% (23·0–48·0)	5·33 (4·59–6·21)	78·7% (68·0–88·1)	0·548 (0·370–0·767)	75·3% (64·7–84·7)
**Fracture of pelvis**						
Females	2·86 (2·01–4·08)	52·8% (30·8–70·3)	8·37 (7·60–9·61)	66·3% (62·4–71·8)	1·42 (1·00–1·97)	64·4% (60·7–69·7)
Males	3·18 (2·42–4·20)	30·5% (12·4–44·3)	10·4 (9·50–11·9)	59·8% (55·7–65·8)	1·80 (1·27–2·46)	57·7% (53·7–63·4)
Both	6·04 (4·44–8·30)	40·2% (20·9–55·2)	18·8 (17·1–21·5)	62·6% (58·9–68·1)	3·22 (2·27–4·43)	60·6% (57·0–65·8)
**Fracture of hip**						
Females	8·14 (6·18–10·6)	107·4% (87·5–120·8)	13·8 (12·6–15·3)	120·3% (112·0–126·3)	1·65 (1·15–2·19)	71·8% (59·9–83·2)
Males	6·11 (4·92–7·53)	76·0% (54·8–90·4)	9·82 (8·89–11·1)	104·2% (91·7–112·3)	1·29 (0·885–1·75)	50·2% (38·1–61·4)
Both	14·2 (11·1–18·1)	92·7% (71·9–106·7)	23·6 (21·6–26·3)	113·3% (102·8–120·1)	2·94 (2·03–3·96)	61·6% (49·6–73·1)
**Fracture of femur, other than femoral neck**						
Females	6·75 (5·26–8·63)	56·7% (41·5–69·5)	21·1 (19·5–23·1)	106·8% (101·6–112·1)	0·890 (0·595–1·27)	99·0% (92·1–105·4)
Males	7·89 (6·48–9·59)	35·1% (24·1–44·2)	22·1 (20·3–23·9)	92·4% (86·8–97·9)	0·961 (0·641–1·37)	83·4% (75·8–90·5)
Both	14·6 (11·9–18·0)	44·3% (31·5–54·6)	43·2 (39·8–46·8)	99·2% (93·7–104·5)	1·85 (1·23–2·64)	90·6% (83·4–97·3)
**Fracture of patella, tibia or fibula, or ankle**						
Females	13·4 (10·8–16·9)	40·9% (34·8–46·2)	127 (118–139)	70·5% (68·2–73·2)	6·52 (4·28–9·44)	69·3% (66·9–72·0)
Males	19·3 (16·0–23·0)	30·7% (25·2–35·6)	172 (161–185)	62·2% (59·9–64·3)	8·99 (5·89–13·0)	61·3% (59·0–63·7)
Both	32·7 (26·9–39·7)	34·7% (29·0–39·4)	300 (279–323)	65·6% (63·3–67·8)	15·5 (10·2–22·6)	64·6% (62·3–66·9)
**Fracture of foot bones except ankle**						
Females	3·90 (2·81–5·29)	26·7% (15·7–33·9)	7·75 (7·03–8·79)	76·9% (72·1–82·3)	0·189 (0·116–0·295)	75·1% (70·6–81·5)
Males	6·78 (5·22–8·67)	18·2% (11·1–23·6)	11·7 (10·6–12·9)	66·4% (61·9–71·5)	0·290 (0·176–0·455)	65·2% (60·6–71·1)
Both	10·7 (8·07–14·0)	21·1% (12·7–26·7)	19·4 (17·8–21·7)	70·4% (66·1–75·6)	0·480 (0·291–0·750)	68·9% (64·6–74·8)

Values in parentheses are 95% uncertainty intervals. Counts are given to three significant figures and percentage changes to one decimal place.

**Table 2:** Global age-standardised rates of incidence, prevalence, and YLDs of fractures by anatomical site and sex in 2019, and percentage change from 1990 to 2019

	**Age standardised incidence rate (per 100 000)**		**Age-standardised prevalence rate (per 100 000)**		**Age-standardised YLDs rate (per 100 000)**	
	2019	Percentage change, 1990–2019 (%)	2019	Percentage change, 1990–2019 (%)	2019	Percentage change, 1990–2019 (%)
**Fractures**						
Females	1943·6 (1739·7 to 2176·2)	−5·4% (−7·1 to −3·9)	4733·5 (4451·4 to 5062·3)	−4·5% (−5·4 to −3·6)	272·5 (189·2 to 376·2)	−6·5% (−7·7 to −5·4)
Males	2619·8 (2406·1 to 2865·7)	−12·0% (−13·6 to −10·4)	6493·6 (6121·8 to 6911·9)	−8·0% (−9·1 to −6·9)	364·8 (249·9 to 510·3)	−9·5% (−10·7 to −8·3)
Both	2296·2 (2091·1 to 2529·5)	−9·6% (−11·1 to −8·1)	5614·3 (5286·1 to 5977·5)	−6·7% (−7·6 to −5·7)	319·0 (220·1 to 442·5)	−8·4% (−9·5 to −7·2)
**Fracture of skull**						
Females	64·2 (47·9 to 88·3)	−12·3% (−21·8 to −7·0)	30·4 (27·0 to 35·5)	−0·7% (−4·2 to 2·4)	2·0 (1·4 to 2·9)	−1·3% (−5·0 to 2·1)
Males	132·7 (104·8 to 168·6)	−15·3% (−21·8 to −10·6)	49·4 (44·1 to 56·4)	−4·8% (−7·6 to −1·9)	3·3 (2·2 to 4·8)	−5·1% (−8·2 to −1·8)
Both	98·9 (77·1 to 128·5)	−14·4% (−21·5 to −9·6)	39·9 (35·7 to 45·8)	−3·3% (−6·3 to −0·4)	2·7 (1·8 to 3·9)	−3·7% (−6·9 to −0·6)
**Fracture of facial bones**						
Females	93·0 (70·4 to 123·9)	−11·2% (−18·4 to −6·5)	19·1 (16·0 to 23·2)	−7·8% (−11·8 to −5·2)	1·2 (0·8 to 1·8)	−8·0% (−12·6 to −5·1)
Males	183·4 (149·4 to 227·7)	−15·3% (−20·3 to −11·6)	35·0 (29·7 to 40·9)	−11·4% (−14·7 to −9·0)	2·3 (1·4 to 3·3)	−11·5% (−15·0 to −8·8)
Both	138·8 (110·6 to 174·8)	−14·0% (−19·6 to −10·2)	27·0 (23·0 to 31·9)	−10·2% (−13·8 to −7·9)	1·7 (1·1 to 2·6)	−10·4% (−14·1 to −7·8)
**Fracture of sternum or fracture of one or more ribs, or both**						
Females	37·6 (25·5 to 56·3)	−2·4% (−6·9 to 2·1)	17·7 (15·6 to 20·3)	−4·3% (−5·8 to −2·8)	1·7 (1·1 to 2·4)	−4·4% (−6·7 to −2·2)
Males	66·6 (48·9 to 91·6)	−6·9% (−10·7 to −2·9)	31·6 (28·3 to 35·8)	−8·6% (−10·1 to −7·1)	3·1 (2·1 to 4·3)	−8·6% (−10·7 to −6·6)
Both	52·2 (37·9 to 74·2)	−5·5% (−9·2 to −1·7)	24·5 (21·9 to 28·1)	−7·0% (−8·4 to −5·6)	2·4 (1·6 to 3·4)	−7·1% (−8·8 to −5·4)
**Fracture of clavicle, scapula, or humerus**						
Females	218·4 (168·1 to 280·9)	−5·8% (−11·1 to −2·6)	83·5 (73·0 to 96·4)	−3·4% (−5·6 to −1·8)	2·8 (1·7 to 4·3)	−3·6% (−6·0 to −1·7)
Males	277·8 (224·9 to 336·8)	−12·7% (−18·5 to −8·7)	102·2 (89·6 to 116·4)	−7·6% (−10·5 to −5·3)	3·4 (2·1 to 5·3)	−7·8% (−10·7 to −5·4)
Both	249·2 (197·1 to 309·6)	−10·0% (−15·4 to −6·4)	93·3 (81·5 to 106·9)	−6·0% (−8·5 to −4·2)	3·1 (1·9 to 4·9)	−6·2% (−8·9 to −4·2)
**Fracture of radius or ulna, or both**						
Females	388·6 (298·1 to 505·7)	−5·0% (−8·9 to −2·4)	76·3 (62·6 to 95·0)	−4·5% (−6·8 to −3·0)	2·5 (1·5 to 3·9)	−4·6% (−6·6 to −2·9)
Males	401·6 (332·5 to 490·9)	−13·1% (−17·1 to −10·3)	82·2 (69·6 to 97·2)	−10·4% (−12·7 to −8·6)	2·8 (1·7 to 4·3)	−9·9% (−12·0 to −7·8)
Both	396·6 (316·0 to 498·3)	−9·5% (−13·4 to −7·0)	79·6 (66·7 to 96·0)	−7·9% (−10·1 to −6·4)	2·7 (1·6 to 4·1)	−7·7% (−9·6 to −6·2)
**Fracture of hand, wrist, or other distal part of hand**						
Females	175·9 (134·0 to 225·6)	−9·0% (−13·6 to −5·8)	225·7 (208·4 to 247·5)	−4·7% (−6·3 to −2·6)	2·8 (1·5 to 5·0)	−4·9% (−6·4 to −2·5)
Males	314·6 (256·4 to 383·4)	−17·0% (−20·3 to −14·4)	362·4 (336·7 to 394·9)	−10·2% (−11·9 to −8·2)	4·6 (2·4 to 8·0)	−10·2% (−11·8 to −7·7)
Both	246·1 (196·5 to 304·4)	−14·4% (−17·9 to −11·7)	293·3 (272·7 to 318·6)	−8·2% (−9·6 to −6·0)	3·7 (2·0 to 6·4)	−8·2% (−9·6 to −5·9)
**Fracture of vertebral column**						
Females	92·2 (68·4 to 125·3)	−5·5% (−14·7 to −0·2)	60·1 (51·5 to 70·9)	1·4% (−2·4 to 3·9)	6·1 (4·1 to 8·7)	0·8% (−3·1 to 3·7)
Males	125·3 (99·2 to 160·1)	−11·4% (−19·0 to −6·6)	73·8 (63·3 to 85·5)	−2·0% (−5·8 to 0·8)	7·6 (5·1 to 10·6)	−2·6% (−6·4 to 0·6)
Both	109·4 (84·4 to 143·7)	−9·2% (−17·1 to −4·4)	67·5 (58·0 to 78·6)	−0·9% (−4·5 to 1·7)	6·9 (4·7 to 9·7)	−1·5% (−5·1 to 1·3)
**Fracture of pelvis**						
Females	70·6 (50·2 to 100·7)	−5·3% (−15·1 to 1·5)	200·6 (182·1 to 232·3)	−6·0% (−8·5 to −1·4)	34·2 (24·0 to 47·3)	−6·1% (−8·5 to −1·6)
Males	81·5 (62·0 to 107·4)	−12·8% (−22·6 to −5·1)	261·6 (238·0 to 297·8)	−8·7% (−11·4 to −4·3)	45·1 (31·7 to 61·7)	−8·8% (−11·3 to −4·8)
Both	76·8 (56·3 to 104·9)	−9·7% (−19·5 to −2·7)	231·0 (209·7 to 263·5)	−7·6% (−10·0 to −3·4)	39·6 (27·9 to 54·5)	−7·7% (−10·0 to −3·5)
**Fracture of hip**						
Females	189·8 (144·2 to 247·2)	2·7% (−3·7 to 6·6)	319·9 (291·1 to 356·2)	7·7% (5·5 to 10·0)	38·4 (26·9 to 51·6)	−11·7% (−15·8 to −7·1)
Males	166·2 (133·2 to 205·8)	0·8% (−7·3 to 6·2)	261·4 (237·3 to 293·5)	10·8% (6·6 to 13·8)	33·7 (23·1 to 45·5)	−15·3% (−20·5 to −9·7)
Both	182·5 (141·9 to 230·9)	0·4% (−6·4 to 4·6)	298·1 (271·6 to 331·6)	7·5% (4·8 to 9·7)	36·8 (25·5 to 49·6)	−14·0% (−18·3 to −9·1)
**Fracture of femur, other than femoral neck**						
Females	166·4 (130·3 to 212·1)	−5·0% (−11·5 to −1·0)	490·9 (453·0 to 537·8)	2·3% (0·6 to 4·0)	20·8 (13·9 to 29·6)	1·3% (−0·7 to 3·1)
Males	203·8 (167·0 to 247·1)	−9·8% (−16·2 to −5·0)	582·8 (537·4 to 630·6)	1·5% (−0·6 to 3·8)	25·2 (16·9 to 36·0)	0·2% (−2·2 to 2·4)
Both	187·2 (152·2 to 229·2)	−8·2% (−14·3 to −3·9)	540·7 (499·2 to 586·7)	1·4% (−0·4 to 3·2)	23·2 (15·5 to 33·0)	0·2% (−2·0 to 2·0)
**Fracture of patella, tibia or fibula, or ankle**						
Females	344·8 (276·0 to 435·5)	−4·0% (−7·3 to −1·9)	3029·8 (2811·9 to 3301·8)	−6·7% (−7·6 to −5·8)	155·3 (102·1 to 224·9)	−6·8% (−7·7 to −5·9)
Males	492·0 (409·8 to 587·7)	−9·0% (−12·5 to −6·2)	4355·7 (4073·5 to 4681·0)	−10·0% (−11·0 to −9·0)	226·3 (148·4 to 328·2)	−10·0% (−11·0 to −8·9)
Both	419·9 (345·8 to 512·0)	−7·2% (−10·4 to −5·0)	3683·5 (3434·4 to 3972·1)	−8·7% (−9·6 to −7·7)	190·4 (125·0 to 276·9)	−8·7% (−9·6 to −7·8)
**Fracture of foot bones except ankle**						
Females	102·2 (73·3 to 138·0)	−9·8% (−16·9 to −5·8)	184·1 (166·7 to 209·6)	−3·8% (−5·9 to −0·2)	4·5 (2·8 to 7·0)	−4·1% (−6·1 to −0·3)
Males	174·2 (134·3 to 222·5)	−15·5% (−20·2 to −12·0)	295·9 (270·5 to 326·8)	−7·1% (−9·5 to −3·9)	7·4 (4·4 to 11·5)	−7·3% (−9·6 to −3·9)
Both	138·7 (104·9 to 182·5)	−13·6% (−19·1 to −10·1)	239·3 (219·0 to 267·6)	−5·9% (−8·0 to −2·8)	5·9 (3·6 to 9·2)	−6·1% (−8·1 to −2·7)

Values in parentheses are 95% uncertainty intervals. Data are given to one decimal place. YLDs=years lived with disability.

**Figure 1:**
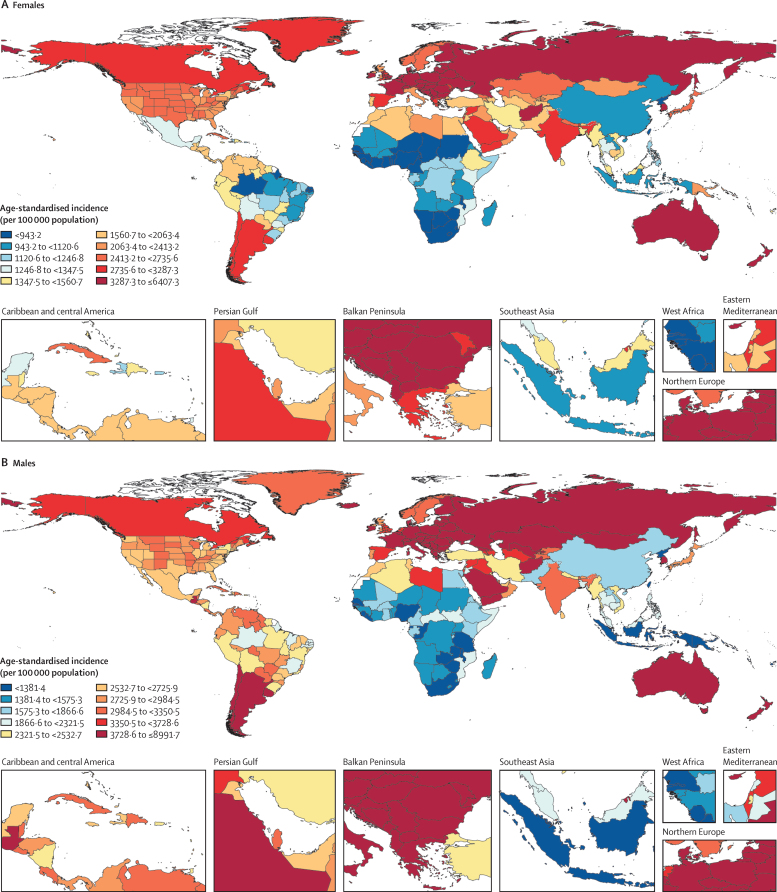
Age-standardised incidence of fractures for females (A) and males (B), 2019

The top three regions by age-standardised incidence rate of fractures in 2019 were Australasia, central Europe, and eastern Europe, and the top three countries and territories were New Zealand, Slovenia, and Australia (appendix pp 10–20). The three regions with the lowest age-standardised incidence rates were southern sub-Saharan Africa, western sub-Saharan Africa, and central sub-Saharan Africa, and the three countries and territories with the lowest rates were North Korea, Liberia, and Zimbabwe (appendix pp 10–20). Maps of the global age-standardised incidence rate of new fractures for females and males for 2019 are shown in figure 1.

**Figure 2:**
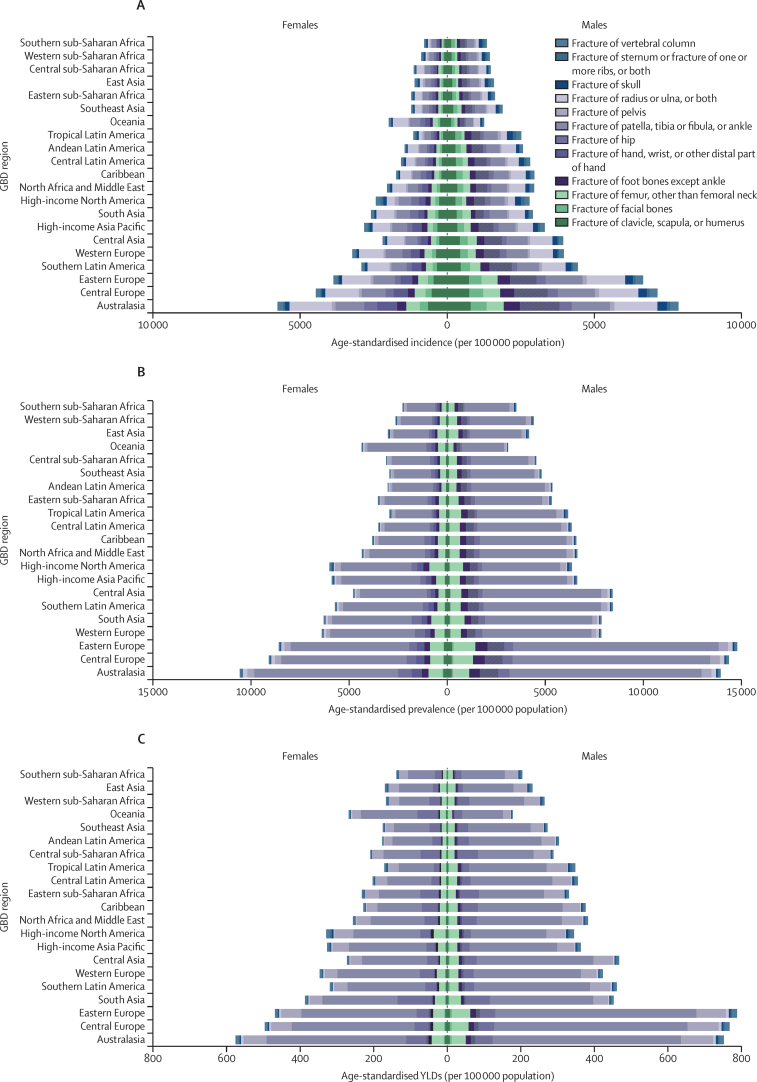
Age-standardised incidence (A), prevalence (B), and YLDs (C) of fractures for each anatomical site, by GBD region and sex, 2019 Regions in each panel are listed from lowest (top) to highest (bottom) age-standardised rate for both sexes combined. GBD=Global Burden of Diseases, Injuries, and Risk Factors Study. YLDs=years lived with disability.

In 2019, the top three anatomical sites for both all-age counts and age-standardised rates of new fractures were fractures of patella, tibia or fibula, or ankle; radius or ulna, or both; and clavicle, scapula, or humerus (Table 1, Table 2). Age-standardised incidence rates for fractures of different anatomical sites in 2019, by GBD region, are shown in figure 2A.

**Figure 3:**
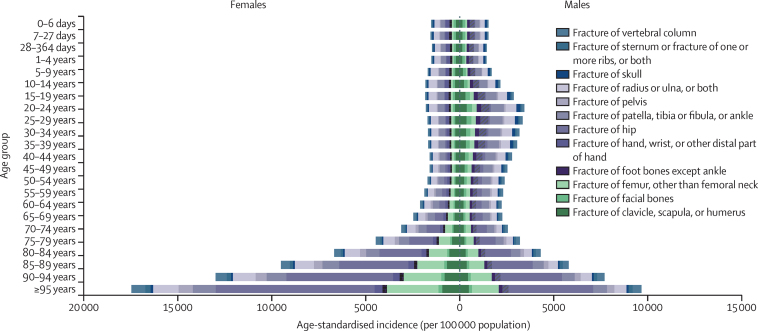
Age-specific incidence rate of fractures for each anatomical site globally, by age group and sex, 2019

Older age groups were the most likely to have new fractures in 2019, with the highest age-specific rates of fracture incidence in those aged 95 years and older, at 15 381·5 incident cases (95% UI 11 245·3–20 651·9) per 100 000 population. We observed a trend of progressively higher incidence rates in older age groups in 2019, which was particularly pronounced in females (figure 3). Although age-standardised and all-age fracture incidence rates were higher among males than females in 2019, for all age groups older than 64 years, females had higher fracture incidence rates than did males. For instance, in 2019, incidence rates among the population aged 95 years and older were 57·4% higher for females (17 465·3 [12 770·5–23 511·1] per 100 000 population) than for males (9672·6 [7099·3–12 971·9] per 100 000 population). By contrast, age-specific incidence rates were more than 50% higher in males than in females in the age 20–24 years group through to the age 40–44 years group in 2019. Incidence data by age group are available on the GBD Results Tool.

In 2019, there were 455 million (95% UI 428–484) prevalent cases of fracture among all age groups combined, an increase of 70·1% (67·5–72·5) since 1990 (table 1; appendix pp 2–9). The age-standardised prevalence rate was 5614·3 (5286·1–5977·5) per 100 000 population, a decrease of 6·7% (5·7–7·6) since 1990 (table 2). Both number and age-standardised rates of prevalent fracture were higher in males than in females. The age-standardised prevalence of fractures decreased significantly for both sexes over the study period (table 2).

In 2019, the top three regions by age-standardised prevalence rate were Australasia, central Europe, and eastern Europe, and the top three countries were Slovenia, New Zealand, and Slovakia; the lowest three regions were southern sub-Saharan Africa, western sub-Saharan Africa, and east Asia, and the lowest three countries were North Korea, Zimbabwe, and Kiribati (appendix pp 10–20).

In 2019, the top three anatomical sites in terms of number of prevalent fractures were patella, tibia or fibula, or ankle; femur, other than femoral neck; and fracture of hand, wrist, or other distal part of hand (table 1). The most frequent sites in terms of age-standardised prevalence rates were fracture of patella, tibia or fibula, or ankle; femur, other than femoral neck; and hip (table 2). Age-standardised prevalence rates by anatomical site and GBD region are shown in figure 2B.

In 2019, there were 25·8 million (95% UI 17·8–35·8) fracture YLDs among all ages, an increase of 65·3% (62·4–68·0) since 1990 (table 1; appendix pp 2–9). The age-standardised rate of YLDs was 319·0 (220·1–442·5) per 100 000 population, a decrease of 8·4% (7·2–9·5) since 1990. Both the total number of YLDs and age-standardised rates of YLDs were higher in males than in females (Table 1, Table 2). The age-standardised rates of fracture YLDs decreased significantly in both sexes over the study period.

In 2019, the three GBD regions with the highest age-standardised rates of fracture YLDs were Australasia, central Europe, and eastern Europe, and the top three countries were Afghanistan, New Zealand, and Slovenia; the lowest three regions were southern sub-Saharan Africa, east Asia, and western sub-Saharan Africa, and the lowest three countries and territories were North Korea, Taiwan (province of China), and Kiribati (appendix pp 10–20).

In 2019, the top three anatomical sites of fracture in terms of number of YLDs and age-standardised YLD rate were fracture of patella, tibia or fibula, or ankle; pelvis; and hip (Table 1, Table 2). In 2019, there were 15·5 million (95% UI 10·2–22·6) YLDs caused by fracture of the patella, tibia or fibula, or ankle, and the age-standardised rate of YLDs was 190·4 (125·0–276·9) per 100 000 population, which were much higher than for the second-leading site, the pelvis (3·22 million [2·27–4·43] YLDs, and age-standardised rate of YLDs of 39·6 [27·9–54·5] per 100 000 population). Age-standardised YLD rates by anatomical site and GBD region are shown in figure 2C.

Among fracture-related Level 3 causes of injury, falls caused the highest age-standardised rates of fracture YLDs in 2019, at 144·6 (95% UI 98·6–207·20) per 100 000 (for more on cause-specific YLD estimates see the GBD Results Tool). In other words, falls were the leading cause of disability due to a fractured bone. The second-leading cause was road injuries (with an age-standardised rate of fracture YLDs of 89·6 [61·1–125·8] per 100 000 population), followed by exposure to mechanical forces (27·3 [17·8–38·2] per 100 000), other unintentional injuries (20·0 [12·8–28·4] per 100 000), and conflict and terrorism (10·4 [5·7–18·4] per 100 000).

## Discussion

From 1990 to 2019, although the global age-standardised rates of fracture incidence, prevalence, and YLDs decreased slightly, the number of incident cases, prevalent cases, and YLDs increased substantially, largely as a result of population growth and ageing. Males also had higher rates of fractures than females throughout the study period for all ages combined, although this pattern was not consistent in all age groups. Abtahi and colleagues^32^ reported that the female-to-male ratio of major osteoporotic fractures among adults aged 50 years and older in Denmark was 2·72–2·93 to 1·00 between 1995 and 2010, and our findings for 2019 agree with these data, with higher fracture incidence rates for females than for males among the older age groups (older than 64 years). In females, we found age-specific incidence rates in 2019 were similarly low from birth through to approximately age 50–54 years, after which point the rates increased steadily through to the oldest age group. By contrast, in males, age-specific fracture incidence rates were higher among those aged 15–44 years than even those aged 70–74 years, possibly because males are more likely to have jobs with high risks of fracture and have more occupational hazards at middle age than females.^33^, ^34^ Furthermore, in many locations, males are more likely to get into road accidents, work outside the home, practise hazardous sports, and engage in violence;^14^, ^17^ these factors could help explain the higher rates of fractures we observed in males than in females.

Despite the relatively high incidence of fractures we observed in males in the middle age groups, fractures in older adults constitute the majority of total fractures. Comparing the differences in incidence rate of fractures between older females and males in 2019, increases in age-specific incidence occurred around age 50–54 years for females, compared with age 65–69 years in males, and substantially increased at age 80 years and older for both sexes; this finding is consistent with the age at which low-trauma osteoporosis is prevalent for female and male populations. A previous study found that the median age of menopause in the US female population was approximately 51 years, suggesting that osteoporotic fractures (also known as fragility fractures) after menopause might be an important contributor to this trend.^35^, ^36^ Expanding access to and making advances in screening, prevention, treatment, and rehabilitation for osteoporosis in older people might decrease the risk of fractures in the future.^37^, ^38^ The age-standardised rate of YLDs for hip fractures decreased by a larger proportion from 1990 to 2019 than for any other anatomical site, which could possibly be attributable to modern advances in traumatology, implantology, and rehabilitation.

To put the disability burden of fractures in the context of other diseases and injuries, for the ten countries and territories with the highest age-standardised rates of fracture YLDs in 2019, fractures would be between the second-leading and fourth-leading cause of YLDs among GBD Level 3 causes (fracture is a nature of injury, rather than a cause of injury, so it is not part of the GBD cause hierarchy).^14^ For instance, in New Zealand, Slovenia, Slovakia, Croatia, and Poland, the age-standardised rate of YLDs from fractures was second only to low back pain in 2019.^14^ These findings show the important role that fracture prevention and improved treatment have in reducing the overall disability burden in countries and territories around the world, but particularly in countries with the highest age-standardised rates of YLDs due to fractures.

Our study also highlights different functional status after a fracture depending on its anatomical site, information that policy makers can use when assessing how to maximise the effect of fracture prevention and treatment programmes. Zura and colleagues^39^ analysed non-union (ie, a bone fracture that does not heal) in 18 human bones. In this study, Zura and colleagues found that non-union had occurred in only 2·1% of fractures of the radius, but had occurred in 14% of fractures of the tibia or fibula. Delayed union and non-union of tibial fractures are common orthopaedic problems,^40^, ^41^, ^42^ sometimes requiring several surgical procedures and debridement, or bone regeneration substitutes (such as bone grafting);^43^ therefore, extending the treatment period and the corresponding disability. For instance, the disability weights of fractures of the pelvis and hip are much higher than for fractures of the radius and ulna.^18^ High rates of non-union and increased duration and severity of treatment for fractures at some anatomical sites lead to increased YLDs and disease burden for some types of fractures, as we observed in our findings, which should be considered when making policy decisions.

Our findings suggest that policy and practice should focus on the following strategies to decrease the incidence and burden of fractures. First, fractures in older people constitute the majority of fractures, so efforts such as expanding screening and treatment of osteoporosis in older people; encouraging exercise and diet that promote bone strength throughout the life course; and providing educational materials, assistive devices, and other products to reduce the risk of falls might help decrease fractures in these age groups. Second, attention should be paid to the occupational hazards that can cause fracture, including making and enforcing policies to provide safe environments for workers. Third, particularly in countries with the highest age-standardised disability burdens due to fractures, such as New Zealand and Slovenia, focusing on evidence-based policies to prevent fractures offers considerable value because fractures contribute so substantially to the overall burden of disability in these countries. Fourth, policy reform and improving car safety standards to decrease fractures caused by motor vehicle road injuries could also be helpful for reducing fracture burden. Finally, the management of different fracture sites should be stratified in locations where this is not already common practice. For example, fractures of the radius or ulna, or both, can be treated with conservative methods (eg, manual reduction and cast external fixation and orthosis). Surgical intervention for fractures of the radius or ulna have been reported to offer no clear benefit over conservative methods, but involves increased costs and risks of major complications.^44^ However, fractures at other anatomical sites such as the patella, tibia or fibula, or ankle and clavicle, scapula, or humerus have a high risk of delayed union or non-union,^41^ accompanied by large economic costs and loss of productivity, and could be treated more aggressively to shorten the treatment period and decrease the risk of delayed union and non-union.

Methodological updates in GBD 2019 enabled us to systematically measure the global, regional, and national burden of fractures by age, sex, and year. However, our study does have similar limitations to other GBD studies. First, data access and quality are heterogeneous among different locations, and data in low-income and middle-income countries are particularly sparse. Furthermore, in some locations, patients might delay or be unable to seek health care for a fracture, leading to under-reporting. However, because few data sources on admissions to hospital due to injuries come from locations with poor access to treatment for fractures, arguably, our statistical model predicts for fractures that warrant health care rather than what would have been observed if we had inpatient data from the poorest countries. Second, patients might have multiple injury outcomes including fractures in different anatomical sites, but we chose to include only the most burdensome of co-occurring injuries because computationally dealing with combinations of multiple injuries in the same individual is complicated. Furthermore, in past iterations of GBD when we tried to assign long-term outcomes to each co-occurring injury using regression methods, we found an implausible proportion of long-term outcomes would be assigned to common but low severity injury outcomes like open wounds and superficial injuries. This decision has led to some underestimation of incidence and short-term disability but not long-term disability, because we assigned all remaining disability at 1 year to the more severe of co-occurring injuries. Third, some fractures (such as vertebral fractures) might not have serious clinical symptoms and might not be detected and, therefore, their incidence could be underestimated. Finally, most prevalence and YLD estimates are from long-term disabling outcomes. These estimates rely on just seven follow-up studies, with only one from outside a high-income country (China). These seven studies were chosen because they report on a wide range of injury outcomes in a comparable manner. In future rounds of GBD, we intend to incorporate many more studies that might only look at follow-up of one particular fracture site. Because such studies heterogeneously report on long-term outcomes, the major task will be to find credible adjustments to approximate common outcomes.

## Data sharing

To download the data used in these analyses, please visit the Global Health Data Exchange GBD 2019 website.

## Declaration of interests

I Filip reports financial support from Avicenna Medical and Clinical Research Institute. R Q Ivers reports grants from National and Medical Research Council of Australia during the conduct of the study. S L James reports employment with Genentech/Roche beginning in 2020, and project funding from Sanofi Pasteur from 2019–2020, outside the submitted work. All other authors declare no competing interests.
