# Supplementary material for: Global, regional, and national burden of bone fractures in 204 countries and territories, 1990–2019: a systematic analysis from the Global Burden of Disease Study 2019
Source: Lancet Healthy Longev. 2021 Sep;2(9):e580–92. doi: 10.1016/S2666-7568(21)00172-0 (PMC8547262; doi:10.1016/S2666-7568(21)00172-0)
Supplement: Supplementary appendix [file mmc1.pdf]

THE LANCET
Healthy Longevity

Supplementary appendix

This appendix formed part of the original submission and has been peer reviewed. We post it as supplied by the authors.

Supplement to: GBD 2019 Fracture Collaborators. Global, regional, and national burden of bone fractures in 204 countries and territories, 1990–2019: a systematic analysis from the Global Burden of Disease Study 2019. *Lancet Healthy Longev* 2021; published online August 20. https://doi.org/10.1016/S2666-7568(21)00172-0.

Supplementary appendix to "Global, regional, and national burden of bone fractures, 1990–2019: a systematic analysis of the Global Burden of Disease Study 2019"

This appendix provides additional tables and author contribution information for "Global, regional, and national burden of bone fractures, 1990–2019: a systematic analysis of the Global Burden of Disease Study 2019."

Table of Contents

Table S1: Number of incident cases, prevalent cases, and years lived with disability of fractures by location in 2019, and percentage change from 1990 to 2019 2

Table S2: Age-standardised rates of incidence, prevalence, and years lived with disability (YLDs) of fractures by location in 2019, and percentage change from 1990 to 2019........ 10

Table S3: short- and long-term disability weights for each of 12 fracture sites, for all locations and years 21

Author Contributions 22

Table S1: Number of incident cases, prevalent cases, and years lived with disability of fractures by location in 2019, and percentage change from 1990 to 2019						
	Number of incident cases (95% UI)		Number of prevalent cases (95% UI)		Number of years lived with disability (95% UI)	
	2019	Percentage change in number of cases between 1990 and 2019 (%)	2019	Percentage change in number of cases between 1990 and 2019 (%)	2019	Percentage change in number of YLDs between 1990 and 2019 (%)
Global	178236070 (162360493 to 196278296)	33.4 (30.1 to 37.0)	454653917 (427883713 to 484415343)	70.1 (67.5 to 72.5)	25843004 (17829430 to 35827330)	65.3 (62.4 to 68.0)
Central Europe, Eastern Europe, and Central Asia	19794759 (17866902 to 22059610)	-19.3 (-21.0 to -17.7)	56424188 (52540963 to 61398317)	-1.5 (-2.7 to -.1)	3028648 (2051349 to 4283957)	-5.3 (-7.3 to -3.5)
Central Asia	2914934 (2609126 to 3243560)	20.6 (17.8 to 23.4)	5722938 (5356452 to 6179873)	43.5 (41.8 to 45.5)	323759 (219577 to 458099)	40.5 (38.2 to 42.9)
Armenia	81839 (73290 to 92139)	-42.9 (-45.6 to -39.9)	223766 (206245 to 243358)	-22.8 (-25.8 to -20.4)	12345 (8614 to 17163)	-27.0 (-31.3 to -23.7)
Azerbaijan	279403 (248270 to 313341)	24.0 (19.1 to 29.3)	616195 (572903 to 667372)	65.7 (62.0 to 70.3)	35059 (23764 to 49511)	61.2 (56.2 to 66.9)
Georgia	144826 (132608 to 158275)	-32.1 (-34.8 to -29.4)	400514 (375780 to 432092)	-13.9 (-15.6 to -11.9)	22180 (15176 to 31020)	-15.1 (-17.2 to -12.3)
Kazakhstan	666231 (604558 to 738213)	3.8 (.3 to 7.3)	1388037 (1295467 to 1501625)	20.8 (18.9 to 22.9)	76835 (51844 to 109237)	16.8 (14.1 to 19.4)
Kyrgyzstan	184169 (163867 to 208078)	16.2 (11.2 to 22.0)	332080 (310587 to 357648)	28.9 (26.6 to 31.4)	18883 (12752 to 26791)	26.3 (22.9 to 29.5)
Mongolia	126221 (114305 to 139035)	79.6 (72.9 to 87.1)	239657 (223283 to 260087)	144.4 (138.8 to 150.3)	13736 (9262 to 19433)	135.2 (128.0 to 142.4)
Tajikistan	260946 (229847 to 294925)	48.2 (42.3 to 53.5)	469624 (429418 to 516032)	89.9 (79.5 to 106.1)	28227 (19909 to 38303)	93.8 (80.4 to 117.0)
Turkmenistan	146302 (130699 to 164310)	36.4 (31.6 to 41.4)	266903 (248122 to 289447)	74.9 (71.5 to 78.8)	15169 (10235 to 21633)	69.8 (64.8 to 74.7)
Uzbekistan	1024996 (910959 to 1150477)	50.5 (44.9 to 55.7)	1786162 (1668240 to 1924292)	86.7 (83.3 to 90.8)	101326 (68142 to 144301)	82.2 (77.7 to 87.0)
Central Europe	6287888 (5659379 to 6973446)	-20.0 (-22.2 to -17.9)	18659278 (17386232 to 20167182)	4.8 (3.2 to 6.6)	994340 (673749 to 1410055)	-.6 (-3.2 to 1.9)
Albania	141526 (126463 to 158481)	-34.6 (-38.2 to -31.2)	372765 (345784 to 404170)	16.2 (12.5 to 20.4)	20015 (13501 to 28625)	9.2 (4.9 to 13.9)
Bosnia and Herzegovina	160240 (143831 to 179632)	-37.3 (-40.0 to -34.5)	531912 (489451 to 585058)	11.1 (6.6 to 16.9)	28900 (20195 to 39672)	4.5 (.0 to 11.7)
Bulgaria	352125 (318350 to 390379)	-34.0 (-36.5 to -31.6)	1142138 (1061778 to 1235926)	-16.4 (-18.0 to -14.8)	61105 (41373 to 86764)	-19.3 (-21.3 to -17.4)
Croatia	278588 (255076 to 305843)	-15.5 (-19.6 to -11.3)	799567 (748388 to 859355)	2.8 (.2 to 5.9)	42870 (29274 to 59918)	.4 (-2.5 to 4.1)
Czech Republic	634308 (574040 to 701241)	-15.7 (-18.7 to -12.5)	1901769 (1778000 to 2054806)	10.5 (8.4 to 12.9)	100606 (68194 to 142731)	4.7 (1.7 to 8.0)
Hungary	547840 (493740 to 609615)	-26.3 (-28.7 to -23.3)	1618908 (1508130 to 1756114)	-8.9 (-11.0 to -6.6)	85956 (58234 to 121998)	-14.6 (-17.9 to -11.5)
Montenegro	33530 (30056 to 37160)	-7.3 (-9.9 to -4.3)	90702 (84207 to 98433)	25.1 (22.7 to 27.5)	4840 (3260 to 6927)	20.8 (18.0 to 23.6)
North Macedonia	104919 (93719 to 117791)	-7.0 (-11.8 to -1.9)	279726 (259189 to 303205)	30.2 (25.1 to 35.2)	15104 (10217 to 21591)	22.9 (17.4 to 28.1)
Poland	2140610 (1912481 to 2388255)	-10.4 (-13.4 to -7.4)	6344804 (5891282 to 6900289)	17.3 (15.1 to 20.0)	337370 (228124 to 479437)	10.4 (6.8 to 14.0)
Romania	977665 (880541 to 1089587)	-35.5 (-38.2 to -32.6)	2950075 (2740170 to 3202335)	-13.8 (-15.5 to -12.2)	157405 (106098 to 223867)	-18.3 (-20.6 to -16.0)
Serbia	428254 (382260 to 480672)	-8.5 (-12.1 to -5.0)	1256500 (1166675 to 1359282)	11.5 (8.7 to 14.8)	67138 (45808 to 94840)	5.8 (2.3 to 10.0)
Slovakia	338923 (306815 to 374929)	-5.5 (-9.1 to -2.0)	935925 (868884 to 1015777)	22.1 (19.9 to 24.8)	49789 (33483 to 70960)	16.8 (13.5 to 20.1)
Slovenia	149360 (135939 to 164804)	-3.2 (-7.0 to .5)	434488 (406755 to 464393)	24.1 (21.7 to 27.0)	23243 (15735 to 32957)	21.7 (18.8 to 24.8)

Eastern Europe	10591937 (9513809 to 11889398)	-25.7 (-27.6 to -23.7)	32041972 (29710072 to 35160997)	-9.7 (-11.0 to -8.3)	1710548 (1155462 to 2420476)	-13.1 (-15.0 to -11.3)
Belarus	486031 (441917 to 531624)	-14.7 (-17.9 to -11.5)	1416233 (1317524 to 1549268)	.7 (-1.5 to 3.0)	75183 (50539 to 106642)	-4.0 (-7.0 to -1.0)
Estonia	57368 (52176 to 63179)	-43.7 (-46.1 to -41.3)	179280 (166824 to 195595)	-29.7 (-31.3 to -28.2)	9497 (6432 to 13476)	-32.7 (-34.7 to -30.8)
Latvia	88049 (80722 to 96016)	-54.2 (-56.1 to -52.2)	280649 (261614 to 304009)	-42.8 (-43.9 to -41.8)	14884 (10075 to 20959)	-45.3 (-46.8 to -43.9)
Lithuania	143303 (131673 to 155958)	-41.5 (-43.8 to -39.2)	454487 (421977 to 491115)	-23.9 (-25.5 to -22.4)	24199 (16415 to 34123)	-26.6 (-28.4 to -24.7)
Moldova	140951 (127692 to 155293)	-39.9 (-42.4 to -37.2)	414663 (387450 to 449965)	-18.9 (-20.6 to -17.0)	22794 (15535 to 32156)	-21.8 (-23.8 to -19.4)
Russia	7485318 (6703752 to 8436100)	-22.7 (-25.3 to -20.2)	22553598 (20928909 to 24738348)	-5.0 (-6.9 to -3.1)	1202961 (813843 to 1699630)	-8.8 (-11.1 to -6.6)
Ukraine	2190919 (1957511 to 2488327)	-32.1 (-34.0 to -30.1)	6743060 (6205349 to 7438119)	-20.3 (-21.5 to -19.3)	361031 (243674 to 514537)	-22.6 (-24.0 to -21.1)
High-income	37339933 (33878564 to 41253752)	17.1 (14.0 to 20.2)	108918997 (101933180 to 117402925)	41.7 (39.7 to 44.1)	5851946 (3965259 to 8225686)	39.2 (37.2 to 41.8)
Australasia	1923618 (1716336 to 2155960)	45.6 (40.6 to 50.7)	4613988 (4310003 to 4970858)	75.8 (73.4 to 79.0)	249377 (168461 to 353245)	75.3 (72.1 to 79.3)
Australia	1590032 (1412391 to 1784157)	49.2 (43.8 to 54.7)	3811852 (3554468 to 4114810)	79.6 (77.0 to 83.1)	206033 (139216 to 292246)	79.3 (75.8 to 83.8)
New Zealand	333586 (301510 to 369674)	30.7 (23.8 to 37.5)	802136 (753999 to 855758)	59.5 (54.8 to 63.8)	43344 (29394 to 61634)	58.7 (54.1 to 63.4)
High-income Asia Pacific	5698731 (5108571 to 6312801)	-.9 (-4.6 to 3.0)	19026088 (17819752 to 20579471)	44.0 (41.5 to 47.4)	1028238 (697377 to 1453617)	39.7 (37.2 to 43.0)
Brunei	15064 (13485 to 16895)	48.8 (43.1 to 54.3)	27733 (25752 to 30355)	96.2 (91.6 to 100.6)	1547 (1041 to 2203)	90.7 (85.1 to 96.3)
Japan	3461359 (3075125 to 3895396)	-4.7 (-8.9 to .5)	12797534 (11923337 to 13865348)	33.1 (30.7 to 36.7)	691968 (468197 to 975188)	30.3 (27.9 to 33.7)
Singapore	172085 (152215 to 195065)	50.7 (43.4 to 58.8)	465880 (433723 to 508938)	133.7 (128.7 to 139.4)	25360 (16967 to 36533)	128.6 (123.0 to 134.7)
South Korea	2050223 (1850082 to 2264682)	2.8 (-2.1 to 8.2)	5734941 (5355432 to 6194973)	69.4 (64.6 to 74.3)	309364 (209194 to 438564)	60.2 (55.1 to 65.5)
High-income North America	10775695 (9646215 to 12020937)	29.9 (24.0 to 36.4)	32731482 (30661592 to 35118807)	51.7 (48.1 to 55.8)	1753417 (1199915 to 2449745)	48.7 (45.0 to 52.7)
Canada	1394350 (1273997 to 1522163)	50.9 (45.2 to 57.0)	3690188 (3497137 to 3931152)	79.8 (76.1 to 83.6)	198200 (134749 to 280184)	78.6 (74.1 to 83.7)
Greenland	1783 (1611 to 1974)	-18.3 (-22.0 to -14.4)	4619 (4312 to 5021)	8.0 (4.4 to 11.9)	262 (180 to 367)	5.8 (2.0 to 9.8)
USA	9379391 (8336633 to 10529652)	27.2 (20.9 to 34.1)	29036156 (27133276 to 31211849)	48.8 (45.1 to 52.9)	1554927 (1064125 to 2169365)	45.6 (41.9 to 49.6)
Southern Latin America	2396035 (2138167 to 2696014)	31.6 (28.4 to 35.0)	5273423 (4947006 to 5667108)	57.5 (55.5 to 59.7)	290472 (196502 to 412729)	50.5 (46.9 to 53.8)
Argentina	1596801 (1422565 to 1802909)	32.2 (28.3 to 36.6)	3417930 (3206255 to 3685483)	50.9 (48.4 to 53.3)	189925 (128515 to 268978)	44.9 (41.1 to 48.4)
Chile	662826 (595768 to 742891)	41.0 (36.4 to 45.9)	1528459 (1428760 to 1638725)	93.4 (90.0 to 96.5)	82599 (55887 to 117753)	82.0 (76.2 to 87.3)
Uruguay	136288 (122387 to 151597)	-3.9 (-8.6 to 1.4)	326769 (307373 to 348800)	12.2 (10.1 to 14.3)	17934 (12169 to 25334)	8.7 (6.3 to 11.4)
Western Europe	16545854 (14868291 to 18464128)	12.5 (9.7 to 15.3)	47274016 (43984310 to 51191525)	30.8 (29.0 to 32.8)	2530443 (1715211 to 3581444)	29.6 (27.6 to 31.7)
Andorra	3454 (3113 to 3823)	73.7 (66.0 to 82.2)	9698 (9009 to 10491)	119.8 (115.7 to 123.5)	531 (361 to 760)	121.5 (115.8 to 127.1)
Austria	365916 (329414 to 407011)	4.1 (.2 to 8.2)	1042510 (967831 to 1131105)	23.7 (21.5 to 26.4)	55433 (37572 to 78636)	23.1 (20.6 to 26.0)
Belgium	581934 (522204 to 647661)	39.7 (35.5 to 44.3)	1591349 (1477751 to 1718428)	51.3 (48.4 to 54.0)	84878 (57671 to 119265)	50.7 (47.2 to 54.1)
Cyprus	47701 (42275 to 53684)	74.1 (67.6 to 81.2)	120080 (110982 to 131204)	117.5 (112.7 to 122.9)	6470 (4362 to 9310)	110.9 (104.7 to 116.7)
Denmark	212580 (188432 to 242571)	-5.1 (-8.9 to -.4)	582334 (536465 to 635231)	9.3 (6.5 to 13.0)	31230 (21193 to 44637)	7.7 (4.6 to 11.5)
Finland	300666 (269054 to 338954)	10.6 (6.9 to 14.6)	875728 (810175 to 949955)	33.7 (31.0 to 36.3)	46385 (31491 to 66236)	32.3 (29.5 to 35.3)

France	3192815 (2875219 to 3533073)	18.7 (14.4 to 22.9)	8535590 (7984112 to 9194404)	36.6 (34.1 to 39.5)	461099 (314123 to 651558)	35.6 (32.6 to 38.8)
Germany	3323389 (3001036 to 3701655)	14.0 (10.0 to 18.6)	9733267 (9037599 to 10564674)	30.1 (27.4 to 32.7)	517818 (351969 to 738235)	28.8 (25.9 to 31.9)
Greece	312829 (279150 to 350619)	-14.5 (-17.3 to -11.3)	1037048 (961530 to 1126442)	11.0 (9.0 to 13.4)	54964 (36935 to 78472)	9.5 (7.1 to 12.1)
Iceland	12487 (10988 to 14214)	35.2 (31.2 to 39.6)	31886 (29445 to 34815)	65.8 (62.9 to 68.9)	1708 (1155 to 2436)	64.8 (61.4 to 68.0)
Ireland	171603 (149948 to 196941)	32.0 (27.1 to 37.3)	434391 (400071 to 475903)	66.6 (62.5 to 70.6)	23273 (15632 to 33401)	64.2 (59.6 to 68.4)
Israel	304139 (260843 to 355956)	79.4 (71.7 to 87.2)	665501 (611127 to 728050)	123.7 (117.6 to 130.9)	36061 (24605 to 51221)	118.6 (112.2 to 127.2)
Italy	1996809 (1747926 to 2262482)	-13.9 (-17.8 to -9.7)	6251065 (5838399 to 6783396)	7.7 (5.8 to 9.8)	331124 (223890 to 471008)	6.3 (4.2 to 8.3)
Luxembourg	25163 (22442 to 28114)	45.4 (39.7 to 51.3)	66490 (61707 to 72132)	57.8 (54.3 to 61.5)	3565 (2422 to 5069)	56.1 (52.4 to 60.6)
Malta	16853 (15026 to 18942)	24.0 (19.5 to 29.0)	50290 (46214 to 55022)	72.0 (68.9 to 75.1)	2678 (1812 to 3839)	67.9 (64.2 to 71.6)
Monaco	983 (883 to 1107)	39.8 (35.5 to 44.3)	3726 (3429 to 4067)	44.6 (42.7 to 46.8)	204 (139 to 288)	43.5 (40.9 to 46.1)
Netherlands	673141 (608822 to 744701)	50.5 (44.0 to 57.1)	1708587 (1604358 to 1832765)	64.5 (61.3 to 68.3)	93622 (63648 to 132261)	65.8 (61.4 to 71.1)
Norway	188581 (163003 to 217817)	26.7 (22.0 to 31.6)	512234 (474451 to 556070)	30.9 (29.3 to 32.5)	28639 (19674 to 40394)	32.6 (30.6 to 34.9)
Portugal	268336 (244232 to 296730)	-20.4 (-24.1 to -16.4)	836101 (783451 to 896239)	2.8 (.0 to 5.5)	44528 (30341 to 62490)	-1.2 (-4.0 to 1.9)
San Marino	892 (791 to 1018)	54.6 (49.4 to 60.2)	2910 (2676 to 3194)	82.6 (78.4 to 85.9)	160 (109 to 227)	80.2 (75.8 to 84.5)
Spain	1504972 (1330495 to 1693888)	12.6 (6.7 to 18.5)	4700196 (4346042 to 5119443)	46.8 (42.7 to 50.9)	250048 (169302 to 358660)	45.1 (40.7 to 49.7)
Sweden	332176 (287229 to 381566)	27.8 (23.0 to 33.2)	983857 (908562 to 1074186)	30.1 (28.1 to 32.5)	54382 (37127 to 76920)	30.0 (27.5 to 33.0)
Switzerland	435006 (392035 to 484693)	8.7 (4.5 to 12.7)	1186425 (1098754 to 1284072)	24.5 (21.8 to 27.2)	64177 (43711 to 91220)	25.1 (22.1 to 28.1)
UK	2259004 (1995935 to 2553043)	22.8 (18.9 to 26.8)	6271540 (5799495 to 6823016)	33.8 (31.5 to 36.3)	335259 (226719 to 477052)	32.2 (29.6 to 34.9)
Latin America and Caribbean	11906737 (10720475 to 13360195)	27.6 (24.3 to 31.3)	28018070 (26204626 to 30011990)	77.0 (73.9 to 80.1)	1603577 (1101157 to 2235754)	68.7 (63.2 to 73.1)
Andean Latin America	1286340 (1160821 to 1421009)	39.6 (30.1 to 49.2)	2527569 (2376031 to 2702125)	106.7 (99.3 to 112.8)	145279 (99266 to 200760)	88.8 (76.1 to 98.2)
Bolivia	225483 (203257 to 249322)	75.6 (68.8 to 82.7)	391770 (369982 to 421227)	115.0 (110.0 to 120.7)	23070 (15770 to 32167)	104.7 (98.0 to 111.7)
Ecuador	392553 (355222 to 429995)	84.9 (78.4 to 91.6)	751635 (707203 to 807204)	132.3 (127.4 to 138.0)	43021 (29164 to 60394)	117.1 (108.4 to 125.3)
Peru	668305 (602799 to 741653)	15.1 (3.8 to 27.1)	1384163 (1293070 to 1484734)	93.0 (83.3 to 100.8)	79188 (54113 to 109417)	72.7 (56.7 to 84.6)
Caribbean	1107264 (1011746 to 1207807)	48.6 (44.7 to 52.6)	2596451 (2448766 to 2759037)	95.1 (89.4 to 103.7)	151227 (105802 to 207739)	93.0 (85.2 to 109.0)
Antigua and Barbuda	2072 (1854 to 2319)	49.7 (43.5 to 56.2)	4572 (4300 to 4887)	94.4 (90.6 to 98.6)	255 (173 to 356)	88.3 (83.2 to 93.3)
The Bahamas	7285 (6628 to 8023)	52.8 (46.7 to 58.8)	16885 (15978 to 17883)	106.8 (102.4 to 111.8)	959 (650 to 1339)	100.8 (95.1 to 107.1)
Barbados	5246 (4773 to 5793)	18.6 (14.3 to 23.1)	14772 (14010 to 15611)	57.1 (54.3 to 60.7)	824 (561 to 1153)	51.3 (47.2 to 55.6)
Belize	9583 (8627 to 10678)	129.1 (115.8 to 142.6)	16834 (15940 to 17760)	218.2 (211.2 to 225.5)	971 (663 to 1354)	207.4 (197.6 to 216.8)
Bermuda	974 (877 to 1088)	18.3 (12.0 to 24.7)	3506 (3308 to 3718)	57.0 (52.6 to 61.9)	195 (133 to 276)	50.2 (45.1 to 55.6)
Cuba	364026 (336272 to 396076)	33.1 (27.3 to 39.7)	914661 (869470 to 963725)	65.7 (61.5 to 69.9)	50708 (34592 to 71438)	58.5 (53.2 to 63.3)
Dominica	1264 (1148 to 1390)	-3.6 (-7.1 to -.3)	3265 (3101 to 3438)	33.4 (31.1 to 35.9)	187 (128 to 258)	30.5 (27.4 to 34.0)
Dominican Republic	250600 (226986 to 277537)	87.5 (80.5 to 94.8)	504645 (479914 to 532325)	146.1 (141.5 to 151.3)	28980 (19626 to 40504)	135.1 (128.4 to 142.3)

Grenada	2368 (2144 to 2623)	38.9 (32.9 to 44.7)	5157 (4877 to 5460)	67.5 (55.8 to 75.5)	294 (200 to 409)	61.1 (49.0 to 70.1)
Guyana	19040 (17485 to 20690)	8.8 (5.6 to 12.4)	36153 (34184 to 38290)	41.2 (38.6 to 44.4)	2104 (1453 to 2931)	36.8 (32.9 to 40.6)
Haiti	243100 (218869 to 269662)	87.8 (81.3 to 94.7)	541684 (470710 to 645983)	189.7 (154.7 to 243.9)	35713 (25015 to 50270)	212.1 (162.7 to 315.0)
Jamaica	57553 (51578 to 64367)	12.8 (8.2 to 18.0)	119605 (112977 to 127495)	57.2 (54.4 to 60.7)	6770 (4587 to 9465)	53.1 (48.9 to 57.5)
Puerto Rico	62916 (57015 to 69921)	5.5 (.9 to 10.5)	214494 (200683 to 229687)	47.6 (43.4 to 53.6)	11767 (8075 to 16526)	40.4 (35.4 to 47.1)
Saint Kitts and Nevis	1043 (929 to 1180)	53.6 (46.6 to 60.4)	2619 (2464 to 2797)	93.1 (89.1 to 97.4)	149 (101 to 209)	90.2 (84.4 to 95.6)
Saint Lucia	3467 (3158 to 3821)	29.7 (23.9 to 35.9)	8589 (8139 to 9100)	107.9 (103.5 to 113.1)	484 (331 to 676)	99.8 (94.2 to 106.1)
Saint Vincent and the Grenadines	2313 (2103 to 2528)	9.6 (5.1 to 14.3)	5364 (5081 to 5685)	70.0 (66.6 to 74.3)	307 (211 to 428)	65.5 (61.1 to 70.7)
Suriname	10992 (9963 to 12087)	59.4 (53.7 to 65.3)	25119 (23804 to 26645)	100.5 (89.2 to 106.8)	1459 (997 to 2042)	93.3 (79.1 to 101.4)
Trinidad and Tobago	24354 (22138 to 26816)	5.7 (1.3 to 10.1)	65550 (62076 to 69534)	73.4 (70.4 to 77.2)	3695 (2508 to 5172)	65.9 (61.0 to 71.3)
Virgin Islands	1561 (1404 to 1737)	.3 (-3.6 to 4.9)	5021 (4751 to 5305)	40.8 (37.5 to 45.5)	283 (194 to 397)	34.1 (30.2 to 39.3)
Central Latin America	5399848 (4851138 to 6064102)	20.9 (16.5 to 24.9)	12019105 (11255932 to 12820532)	67.0 (62.8 to 70.4)	684089 (472719 to 956823)	56.9 (48.5 to 62.2)
Colombia	1063516 (955049 to 1184496)	-3.8 (-13.5 to 2.9)	2540236 (2380376 to 2703524)	53.0 (49.3 to 56.7)	141168 (97529 to 196910)	43.4 (38.0 to 48.6)
Costa Rica	113217 (101705 to 126749)	46.5 (40.4 to 52.7)	251687 (235719 to 269109)	114.0 (110.3 to 117.7)	13834 (9391 to 19382)	104.8 (100.1 to 109.7)
El Salvador	152985 (137831 to 171452)	-25.4 (-34.7 to -14.4)	393576 (344295 to 464298)	3.5 (-9.1 to 17.0)	23065 (16502 to 31247)	-13.4 (-31.3 to 4.1)
Guatemala	506221 (453927 to 568103)	89.4 (63.4 to 113.9)	852327 (779766 to 946107)	122.1 (92.5 to 146.9)	50324 (35125 to 69512)	93.1 (52.4 to 126.9)
Honduras	234466 (210325 to 264048)	93.1 (84.8 to 101.9)	374162 (350361 to 401628)	148.7 (143.0 to 155.6)	21864 (15114 to 30328)	141.8 (133.8 to 151.5)
Mexico	2380433 (2102206 to 2714491)	20.2 (16.1 to 24.5)	5546900 (5155717 to 5980811)	65.3 (62.6 to 67.8)	317891 (217533 to 446096)	59.5 (56.7 to 62.4)
Nicaragua	134703 (117675 to 153527)	40.3 (34.2 to 46.2)	309365 (261764 to 384769)	35.4 (14.7 to 59.5)	18244 (12933 to 25364)	14.3 (-11.8 to 42.8)
Panama	91978 (82199 to 103930)	60.9 (55.0 to 66.0)	187728 (175416 to 202149)	98.1 (88.9 to 103.1)	10436 (7068 to 14638)	88.5 (76.7 to 95.4)
Venezuela	722329 (645124 to 808595)	29.5 (24.1 to 34.9)	1563125 (1469341 to 1668827)	89.4 (85.6 to 93.4)	87263 (60041 to 122393)	80.5 (74.9 to 86.1)
Tropical Latin America	4113285 (3667401 to 4695357)	28.7 (24.3 to 33.3)	10874946 (10057537 to 11837923)	78.8 (75.0 to 82.8)	622982 (423725 to 886085)	73.4 (69.8 to 77.5)
Brazil	3943807 (3511134 to 4506119)	27.6 (23.1 to 32.2)	10555708 (9756927 to 11494875)	78.0 (74.2 to 82.1)	604794 (411180 to 860674)	72.7 (69.0 to 76.8)
Paraguay	169478 (150688 to 191487)	64.4 (58.5 to 70.7)	319238 (296952 to 343166)	109.1 (105.5 to 113.1)	18188 (12328 to 25851)	101.4 (96.0 to 106.8)
North Africa and Middle East	15532546 (14063750 to 17308017)	75.6 (68.8 to 82.9)	30779714 (27441310 to 35188326)	123.5 (105.1 to 135.0)	1815526 (1274100 to 2483183)	107.4 (79.6 to 123.5)
Afghanistan	1527475 (1255804 to 1857309)	399.7 (353.5 to 450.9)	2626109 (1708212 to 4422401)	104.9 (33.3 to 257.4)	195976 (115632 to 343522)	82.1 (9.1 to 264.1)
Algeria	850510 (769560 to 950362)	47.5 (41.2 to 53.8)	1806641 (1680128 to 1960092)	112.1 (104.8 to 121.2)	102209 (70268 to 142372)	104.5 (96.7 to 114.8)
Bahrain	28208 (25088 to 31790)	176.5 (162.2 to 191.2)	69623 (64335 to 76741)	326.8 (313.9 to 340.8)	3844 (2595 to 5523)	301.5 (283.3 to 320.6)
Egypt	1720662 (1529046 to 1947024)	91.9 (83.4 to 100.5)	3027464 (2830781 to 3270564)	110.9 (105.9 to 116.0)	172352 (117078 to 243451)	101.6 (94.4 to 108.9)
Iran	1666905 (1488444 to 1880715)	-21.7 (-28.7 to -13.8)	4058322 (3723621 to 4460715)	50.9 (41.0 to 60.3)	226857 (160219 to 311577)	36.0 (21.9 to 48.0)
Iraq	1286531 (1165175 to 1430216)	92.7 (80.5 to 104.2)	3202530 (2527652 to 4216648)	120.3 (109.4 to 131.2)	197624 (140324 to 280487)	101.0 (72.2 to 122.1)

Jordan	220876 (194696 to 252877)	196.2 (182.0 to 211.5)	357603 (330362 to 393777)	285.3 (274.8 to 296.6)	20006 (13448 to 28711)	266.9 (252.5 to 281.4)
Kuwait	102008 (90395 to 115480)	-7.9 (-25.5 to 15.2)	223624 (205289 to 246149)	191.7 (175.0 to 206.4)	12352 (8336 to 17601)	159.8 (128.2 to 183.0)
Lebanon	105091 (93022 to 119267)	-7.0 (-23.6 to 12.3)	303175 (241904 to 428518)	32.9 (10.2 to 60.1)	16630 (11426 to 24301)	13.0 (-15.7 to 43.1)
Libya	200243 (179156 to 226438)	120.6 (100.7 to 143.2)	383282 (342953 to 434292)	173.4 (157.8 to 194.8)	22447 (16041 to 30613)	168.4 (151.7 to 192.7)
Morocco	832496 (751277 to 921921)	37.9 (32.3 to 43.8)	1728622 (1611795 to 1880321)	82.6 (77.1 to 88.8)	99069 (68153 to 139021)	73.8 (66.8 to 80.5)
Oman	124140 (110625 to 140561)	142.1 (128.4 to 157.7)	218632 (201601 to 240551)	189.3 (180.6 to 200.2)	12100 (8092 to 17457)	176.5 (164.6 to 189.7)
Palestine	112552 (100216 to 127688)	47.8 (27.4 to 72.2)	315073 (240934 to 425901)	196.5 (170.2 to 234.1)	19476 (13753 to 27788)	177.1 (136.0 to 229.5)
Qatar	96283 (85401 to 109363)	527.1 (498.8 to 560.9)	163302 (148343 to 182963)	567.4 (546.4 to 586.3)	8978 (5988 to 12959)	534.3 (504.9 to 560.6)
Saudi Arabia	2009631 (1801318 to 2260835)	190.9 (173.5 to 209.8)	3558291 (3212600 to 4007087)	278.3 (261.4 to 295.9)	195043 (129906 to 283267)	255.1 (236.9 to 273.9)
Sudan	525220 (467561 to 598958)	4.2 (-15.4 to 29.0)	1089414 (960181 to 1289548)	119.6 (106.9 to 136.3)	68530 (48508 to 93594)	109.1 (91.4 to 127.8)
Syria	451179 (391138 to 521857)	83.5 (63.8 to 105.4)	1294835 (966963 to 1763118)	271.1 (192.9 to 387.1)	77153 (54577 to 108936)	259.5 (182.9 to 390.2)
Tunisia	240361 (216043 to 268353)	35.9 (30.3 to 42.3)	553531 (517625 to 600331)	99.8 (94.9 to 105.0)	30269 (20530 to 43287)	88.7 (82.0 to 95.7)
Turkey	1718251 (1533671 to 1927176)	45.6 (38.1 to 53.3)	3963031 (3688370 to 4320668)	118.5 (111.6 to 127.7)	218449 (149165 to 309043)	102.6 (93.7 to 113.3)
United Arab Emirates	232420 (207129 to 259296)	370.9 (344.2 to 400.2)	539678 (500222 to 589309)	584.7 (568.8 to 603.4)	30675 (20562 to 43596)	560.7 (535.5 to 584.6)
Yemen	1465726 (1176572 to 1814396)	446.8 (332.2 to 584.7)	1265660 (1104790 to 1484373)	226.7 (202.6 to 259.4)	83643 (59280 to 116098)	240.6 (208.1 to 285.7)
South Asia	48309459 (43196814 to 53770768)	78.5 (72.2 to 85.2)	109739365 (102850898 to 116558586)	130.1 (125.8 to 134.0)	6520403 (4538695 to 8955882)	122.6 (118.9 to 126.6)
Bangladesh	2845836 (2544241 to 3184699)	72.6 (65.8 to 80.4)	6525136 (6096789 to 6959069)	139.6 (133.5 to 145.3)	382623 (261527 to 534958)	129.0 (121.6 to 136.7)
Bhutan	18359 (16592 to 20212)	54.1 (47.1 to 61.4)	38779 (36555 to 41240)	108.0 (103.2 to 113.5)	2284 (1568 to 3164)	98.0 (92.2 to 104.8)
India	40796943 (36449124 to 45442178)	74.3 (67.4 to 81.8)	94349164 (88453663 to 100355488)	126.9 (122.1 to 131.3)	5607167 (3905271 to 7716833)	119.6 (115.5 to 123.8)
Nepal	748223 (673905 to 830546)	73.3 (68.0 to 79.0)	1511372 (1410696 to 1625843)	115.8 (110.9 to 121.9)	91280 (63932 to 124365)	110.7 (104.3 to 119.4)
Pakistan	3900098 (3472174 to 4413567)	148.7 (141.2 to 156.2)	7314915 (6852436 to 7794170)	173.4 (168.5 to 179.4)	437051 (300742 to 600344)	167.1 (159.9 to 176.3)
Southeast Asia, East Asia, and Oceania	32418280 (29278153 to 35891118)	52.9 (45.4 to 60.9)	95216398 (88991691 to 101401467)	118.1 (112.4 to 124.1)	5381454 (3656207 to 7631485)	102.8 (95.6 to 109.9)
East Asia	21818929 (19504771 to 24248696)	66.6 (55.0 to 78.4)	69456564 (64717254 to 74183295)	135.6 (127.9 to 143.9)	3877603 (2613426 to 5516638)	118.0 (110.3 to 126.5)
China	21272704 (18994803 to 23683515)	69.6 (57.5 to 81.8)	67849629 (63202008 to 72505607)	139.3 (131.3 to 147.8)	3788940 (2553189 to 5390512)	121.0 (113.1 to 129.8)
North Korea	254345 (232869 to 276431)	17.1 (12.3 to 23.2)	698436 (660507 to 734419)	57.1 (53.2 to 61.2)	40328 (27295 to 56216)	54.8 (49.8 to 60.2)
Taiwan (Province of China)	291880 (268691 to 316861)	-13.4 (-17.9 to -8.9)	908499 (859211 to 961574)	33.1 (28.6 to 37.9)	48335 (32488 to 68419)	26.4 (21.3 to 31.2)
Oceania	207313 (186275 to 232366)	139.9 (132.5 to 147.3)	359804 (333593 to 388009)	170.9 (165.5 to 176.3)	21560 (15095 to 29937)	167.6 (160.8 to 174.3)
American Samoa	634 (567 to 725)	12.8 (8.7 to 17.1)	1553 (1435 to 1684)	60.0 (54.8 to 66.7)	90 (64 to 124)	56.3 (50.0 to 64.8)
Cook Islands	223 (200 to 253)	-1.5 (-6.3 to 3.2)	735 (683 to 797)	51.3 (47.6 to 56.4)	41 (28 to 57)	44.3 (39.1 to 50.5)
Micronesia (Federated States of)	1480 (1328 to 1659)	12.7 (8.6 to 16.9)	2867 (2667 to 3100)	44.1 (40.8 to 47.9)	167 (115 to 235)	39.7 (35.0 to 44.6)

Fiji	11680 (10341 to 13256)	24.6 (20.7 to 28.5)	24546 (22713 to 26645)	64.1 (60.8 to 67.3)	1427 (978 to 2002)	60.6 (55.6 to 65.7)
Guam	1935 (1731 to 2198)	21.0 (16.2 to 25.6)	5404 (5016 to 5845)	72.6 (68.9 to 77.1)	310 (212 to 435)	67.9 (62.8 to 74.3)
Kiribati	1287 (1141 to 1473)	74.1 (67.6 to 80.9)	2324 (2166 to 2508)	93.4 (89.5 to 97.9)	139 (97 to 192)	92.3 (84.9 to 99.4)
Marshall Islands	788 (709 to 886)	38.9 (33.8 to 44.4)	1461 (1363 to 1577)	89.0 (85.2 to 92.7)	86 (59 to 120)	85.0 (78.9 to 91.7)
Nauru	125 (111 to 143)	18.0 (13.9 to 22.4)	224 (207 to 245)	21.4 (18.9 to 23.9)	13 (9 to 19)	20.1 (15.8 to 24.3)
Niue	20 (18 to 22)	-16.5 (-19.7 to -13.4)	59 (55 to 64)	2.7 (.6 to 5.3)	3 (2 to 5)	.4 (-2.8 to 4.3)
Northern Mariana Islands	594 (537 to 663)	-10.9 (-15.8 to -6.0)	1742 (1618 to 1901)	46.9 (42.4 to 53.5)	99 (67 to 140)	41.2 (35.9 to 48.6)
Palau	335 (304 to 372)	29.8 (24.9 to 34.6)	921 (849 to 1009)	91.4 (87.4 to 96.3)	52 (36 to 73)	85.9 (79.6 to 92.5)
Papua New Guinea	157374 (141574 to 176348)	185.4 (174.8 to 196.2)	264746 (245667 to 285059)	216.0 (209.1 to 223.1)	15976 (11191 to 22177)	211.9 (201.9 to 221.5)
Samoa	3139 (2791 to 3575)	33.8 (27.0 to 39.9)	6097 (5673 to 6558)	79.8 (74.7 to 87.2)	356 (252 to 491)	77.1 (70.4 to 86.7)
Solomon Islands	12602 (11167 to 14211)	124.2 (115.1 to 132.8)	20164 (18473 to 22179)	152.2 (146.3 to 158.5)	1187 (818 to 1672)	146.8 (138.8 to 155.0)
Tokelau	15 (13 to 17)	-3.1 (-6.3 to .4)	37 (34 to 40)	9.1 (7.0 to 11.8)	2 (1 to 3)	6.2 (2.6 to 9.6)
Tonga	1323 (1161 to 1513)	10.4 (6.3 to 14.8)	2511 (2331 to 2723)	35.5 (32.2 to 38.7)	147 (101 to 206)	32.8 (28.5 to 37.2)
Tuvalu	126 (113 to 143)	47.6 (42.2 to 53.2)	301 (279 to 326)	55.0 (51.8 to 58.6)	18 (12 to 25)	50.5 (45.4 to 56.0)
Vanuatu	3844 (3419 to 4344)	114.3 (107.5 to 122.3)	7117 (6645 to 7617)	154.4 (149.1 to 160.5)	427 (300 to 590)	150.6 (142.7 to 158.8)
Southeast Asia	10392038 (9475762 to 11439351)	29.6 (25.0 to 34.2)	25400031 (23942420 to 27063426)	80.7 (74.0 to 85.1)	1482292 (1018697 to 2043987)	71.0 (60.2 to 77.1)
Cambodia	304279 (275191 to 336599)	70.5 (59.2 to 81.2)	704113 (602543 to 903585)	79.4 (32.5 to 127.9)	43902 (30525 to 64156)	51.0 (-.7 to 111.5)
Indonesia	3011634 (2678407 to 3397860)	22.6 (19.2 to 26.1)	8100600 (7623304 to 8618263)	54.3 (50.6 to 57.2)	484643 (335343 to 669888)	49.8 (44.0 to 53.3)
Laos	105790 (96082 to 116641)	32.4 (6.8 to 53.2)	198428 (187611 to 210821)	87.6 (82.2 to 92.0)	11999 (8241 to 16619)	81.1 (72.4 to 88.3)
Malaysia	558169 (509296 to 615413)	94.9 (87.5 to 102.7)	1225085 (1161157 to 1295836)	145.5 (141.6 to 149.5)	68557 (46315 to 96268)	132.6 (125.1 to 139.9)
Maldives	7495 (6713 to 8415)	133.0 (118.7 to 148.5)	14413 (13450 to 15479)	230.4 (220.3 to 241.8)	804 (545 to 1145)	207.5 (192.5 to 221.7)
Mauritius	15534 (14080 to 17234)	38.3 (32.0 to 45.4)	50222 (47160 to 53701)	102.1 (96.5 to 108.9)	2825 (1935 to 3977)	93.8 (86.9 to 101.4)
Myanmar	1020670 (934356 to 1118710)	19.7 (15.0 to 24.0)	2294842 (2103067 to 2544043)	61.1 (47.3 to 71.1)	140098 (99889 to 188876)	55.9 (33.9 to 70.1)
Philippines	1752300 (1578007 to 1962548)	51.7 (44.3 to 58.8)	3408254 (3187194 to 3668466)	106.6 (96.7 to 112.9)	201876 (138326 to 277111)	97.6 (83.0 to 106.0)
Seychelles	1262 (1142 to 1407)	44.5 (39.0 to 50.0)	3612 (3397 to 3843)	90.2 (86.7 to 93.4)	208 (142 to 290)	84.3 (79.2 to 90.0)
Sri Lanka	442811 (405727 to 484242)	-37.1 (-47.3 to -24.5)	1280661 (1139705 to 1466528)	86.3 (74.0 to 101.5)	72655 (52634 to 98166)	67.0 (52.3 to 84.5)
Thailand	1260568 (1158306 to 1366392)	10.0 (4.6 to 15.7)	3670316 (3463565 to 3899796)	72.6 (67.8 to 77.9)	201362 (136623 to 285258)	60.4 (54.3 to 66.5)
Timor-Leste	18756 (16965 to 20693)	-22.5 (-36.9 to -4.3)	53926 (44048 to 67932)	60.7 (52.5 to 69.9)	3580 (2519 to 5171)	36.1 (17.7 to 52.4)
Vietnam	1879156 (1715622 to 2047821)	69.9 (62.3 to 77.7)	4362282 (4132472 to 4605329)	137.5 (132.4 to 142.9)	247842 (169390 to 348196)	122.3 (114.3 to 130.3)
Sub-Saharan Africa	12934355 (11629104 to 14515566)	20.4 (3.8 to 38.8)	25557184 (23432919 to 28377586)	109.2 (103.7 to 114.1)	1641449 (1163699 to 2227430)	99.4 (89.3 to 106.8)
Central Sub-Saharan Africa	1701700 (1538078 to 1891783)	96.5 (84.5 to 108.9)	3215063 (2842393 to 3759467)	157.0 (146.7 to 171.6)	214640 (153880 to 295767)	151.4 (140.2 to 167.2)
Angola	371904 (331892 to 417561)	18.1 (-5.8 to 47.1)	875724 (730212 to 1101298)	105.7 (93.1 to 120.1)	59625 (41975 to 85947)	74.4 (46.9 to 97.9)

Central African Republic	82765 (73403 to 94197)	170.7 (145.2 to 202.1)	148836 (126648 to 182298)	216.0 (168.9 to 291.2)	10920 (7629 to 16433)	272.1 (193.4 to 429.4)
Congo (Brazzaville)	60605 (54287 to 67491)	105.3 (98.8 to 112.1)	153421 (131855 to 185702)	233.3 (187.9 to 303.2)	10189 (7224 to 14391)	258.2 (196.0 to 367.3)
DR Congo	1145892 (1032776 to 1277225)	142.5 (133.3 to 152.3)	1968195 (1771251 to 2228611)	180.8 (157.4 to 219.7)	129782 (93185 to 177839)	198.5 (163.1 to 264.7)
Equatorial Guinea	17459 (15517 to 19701)	240.8 (224.3 to 258.8)	25218 (23760 to 26701)	222.9 (215.4 to 229.2)	1507 (1032 to 2084)	207.1 (192.4 to 219.9)
Gabon	23075 (20865 to 25555)	70.3 (65.3 to 75.4)	43670 (41282 to 45951)	87.1 (85.0 to 89.2)	2617 (1810 to 3605)	82.4 (77.1 to 87.2)
Eastern Sub-Saharan Africa	5581264 (4977856 to 6304646)	-21.3 (-36.5 to -2.5)	11140231 (9935329 to 12906970)	87.9 (78.3 to 96.1)	725824 (514841 to 989678)	72.6 (57.3 to 85.1)
Burundi	190027 (168836 to 214705)	111.1 (103.1 to 119.2)	764230 (557491 to 1051565)	443.0 (298.3 to 656.9)	57444 (37415 to 92160)	561.4 (350.3 to 963.6)
Comoros	10110 (9030 to 11446)	58.7 (52.9 to 64.9)	25749 (24368 to 27319)	94.5 (92.0 to 96.7)	1598 (1110 to 2206)	90.5 (85.5 to 94.9)
Djibouti	15049 (13415 to 17114)	65.9 (45.0 to 90.1)	34893 (32521 to 37620)	226.3 (216.2 to 240.9)	2178 (1520 to 2974)	213.0 (195.3 to 231.9)
Eritrea	83079 (73427 to 94580)	-80.5 (-85.8 to -71.1)	330042 (243679 to 480499)	8.0 (-2.9 to 22.9)	23324 (15345 to 36768)	-14.4 (-29.9 to 4.8)
Ethiopia	1615254 (1439233 to 1817260)	-64.5 (-73.2 to -52.0)	2926051 (2562371 to 3587916)	37.5 (22.7 to 59.1)	191295 (133619 to 270819)	20.2 (.4 to 44.5)
Kenya	761375 (677707 to 858335)	119.3 (115.0 to 123.8)	1231265 (1155909 to 1307029)	165.8 (161.6 to 171.5)	75458 (52211 to 103033)	164.0 (158.7 to 173.0)
Madagascar	325600 (288033 to 371502)	116.4 (108.2 to 125.1)	612627 (572058 to 652393)	114.7 (111.0 to 118.3)	38455 (26471 to 53536)	114.9 (108.5 to 121.4)
Malawi	199934 (175484 to 229870)	100.1 (93.5 to 107.4)	353203 (329751 to 375877)	94.7 (91.3 to 98.0)	21827 (15088 to 30334)	93.4 (87.3 to 99.1)
Mozambique	434758 (384462 to 497063)	64.9 (30.0 to 102.1)	910239 (810927 to 1068964)	53.7 (22.1 to 83.4)	57795 (40668 to 79666)	28.6 (-8.4 to 65.7)
Rwanda	156540 (138088 to 179053)	-32.5 (-46.4 to -13.5)	607260 (467820 to 822389)	176.2 (114.8 to 269.8)	41309 (27253 to 64937)	183.0 (109.8 to 312.5)
Somalia	322119 (279430 to 374734)	49.1 (23.4 to 83.8)	534051 (457106 to 683124)	185.9 (108.7 to 243.3)	37271 (26114 to 53812)	179.8 (87.8 to 258.9)
South Sudan	138191 (122488 to 157146)	85.0 (71.7 to 100.4)	299190 (256986 to 372349)	77.0 (65.6 to 93.2)	20653 (14425 to 29522)	82.4 (68.4 to 100.6)
Tanzania	650895 (574443 to 748370)	118.5 (112.2 to 125.0)	1207411 (1131072 to 1283336)	127.3 (124.5 to 130.0)	74545 (51459 to 103831)	126.1 (120.1 to 132.1)
Uganda	476291 (415468 to 546478)	101.4 (85.2 to 117.4)	953911 (849763 to 1125574)	71.2 (40.2 to 100.5)	61246 (42913 to 83419)	53.0 (16.7 to 89.8)
Zambia	197581 (174146 to 225708)	130.7 (121.1 to 140.2)	341201 (318365 to 363770)	143.9 (139.9 to 147.9)	20847 (14330 to 28867)	138.3 (130.4 to 146.1)
Southern Sub-Saharan Africa	830314 (745531 to 938788)	27.8 (24.3 to 31.3)	1899070 (1778795 to 2025840)	52.3 (48.5 to 54.8)	114259 (78848 to 157667)	47.1 (41.5 to 50.5)
Botswana	26351 (23627 to 29432)	92.0 (84.0 to 100.7)	55789 (52382 to 59374)	144.4 (140.9 to 148.4)	3316 (2275 to 4649)	135.7 (128.3 to 143.1)
Eswatini	12272 (10970 to 13743)	58.2 (52.4 to 64.8)	23618 (22172 to 25092)	94.9 (91.5 to 98.2)	1422 (979 to 1983)	86.6 (80.0 to 93.1)
Lesotho	24316 (21958 to 26978)	37.6 (32.0 to 43.4)	51035 (47951 to 54324)	63.6 (60.7 to 66.1)	3087 (2132 to 4259)	57.9 (52.8 to 62.7)
Namibia	26443 (23713 to 29735)	81.3 (75.9 to 86.5)	61156 (56267 to 67539)	59.4 (38.4 to 75.9)	3752 (2679 to 5066)	41.3 (14.0 to 64.1)
South Africa	596142 (533233 to 674099)	18.5 (14.4 to 22.8)	1465381 (1370949 to 1567839)	47.6 (43.3 to 50.4)	87578 (60267 to 120432)	42.2 (36.9 to 45.9)
Zimbabwe	144791 (129739 to 162449)	55.5 (49.2 to 63.6)	242091 (227836 to 256827)	61.7 (59.6 to 64.0)	15105 (10359 to 20758)	62.2 (57.1 to 67.8)
Western Sub-Saharan Africa	4821077 (4319249 to 5430304)	125.4 (119.4 to 131.3)	9302820 (8733852 to 9932408)	145.6 (141.2 to 153.3)	586726 (409695 to 793906)	144.9 (139.2 to 154.5)
Benin	125677 (111141 to 141110)	163.3 (154.6 to 172.8)	240564 (227941 to 253770)	166.9 (163.3 to 170.2)	15098 (10464 to 20855)	165.7 (157.8 to 173.2)
Burkina Faso	273945 (244574 to 308713)	212.8 (192.6 to 233.6)	462813 (437322 to 489377)	165.6 (162.5 to 168.9)	29388 (20463 to 40583)	167.3 (159.9 to 174.8)
Cameroon	354855 (317154 to 399706)	227.1 (212.2 to 244.2)	691423 (653256 to 732817)	203.2 (198.2 to 209.9)	43149 (29945 to 58540)	199.8 (189.1 to 211.1)

Cape Verde	6401 (5775 to 7131)	86.2 (77.7 to 94.8)	15333 (14496 to 16262)	123.5 (119.8 to 127.2)	911 (625 to 1260)	113.8 (107.0 to 120.6)
Chad	171871 (154008 to 193398)	93.3 (71.3 to 117.8)	329357 (300237 to 373313)	127.8 (109.4 to 142.5)	21697 (15408 to 29403)	111.8 (87.0 to 133.6)
Cote d'Ivoire	268084 (238672 to 300973)	116.2 (110.4 to 122.4)	555794 (525314 to 588129)	149.1 (145.4 to 154.2)	34899 (24473 to 47717)	150.5 (142.5 to 159.3)
The Gambia	21048 (18599 to 23669)	135.4 (128.4 to 142.8)	42476 (40129 to 44993)	146.7 (132.2 to 155.4)	2658 (1860 to 3619)	136.9 (116.1 to 151.4)
Ghana	372869 (331048 to 420334)	147.8 (139.9 to 157.2)	858851 (804757 to 916123)	180.5 (177.3 to 183.3)	52826 (36574 to 73394)	174.3 (166.7 to 181.1)
Guinea	128184 (114108 to 144427)	110.4 (103.5 to 117.8)	266251 (251898 to 281753)	106.2 (103.3 to 109.7)	16807 (11736 to 22866)	104.5 (98.6 to 110.7)
Guinea-Bissau	21154 (18684 to 23780)	85.9 (79.5 to 92.7)	45559 (42688 to 48842)	95.3 (90.7 to 102.9)	2906 (2050 to 3966)	96.5 (89.1 to 107.4)
Liberia	41952 (37124 to 47144)	-77.2 (-83.1 to -66.8)	131436 (109200 to 173795)	97.5 (63.9 to 139.2)	8908 (6209 to 12982)	74.5 (32.8 to 126.0)
Mali	258521 (229426 to 293254)	127.6 (118.7 to 136.4)	454608 (412890 to 520284)	164.7 (145.3 to 201.1)	30122 (21449 to 40873)	172.0 (145.8 to 217.3)
Mauritania	44347 (39380 to 49887)	71.0 (64.5 to 78.2)	99941 (94376 to 105948)	81.8 (79.6 to 83.8)	6187 (4249 to 8526)	77.2 (72.4 to 81.9)
Niger	234495 (209743 to 263207)	168.1 (160.5 to 176.3)	369324 (349467 to 390999)	170.2 (166.9 to 174.4)	23705 (16493 to 32362)	167.8 (159.3 to 176.9)
Nigeria	2185251 (1944960 to 2473907)	146.9 (139.0 to 155.6)	4015346 (3768588 to 4281079)	132.0 (126.6 to 141.7)	251224 (174451 to 342678)	134.1 (126.7 to 147.9)
São Tomé and PrÍncipe	2585 (2324 to 2876)	88.0 (80.7 to 95.0)	5402 (5099 to 5726)	108.5 (105.2 to 112.4)	328 (226 to 456)	103.4 (97.3 to 109.5)
Senegal	145411 (128903 to 162753)	91.5 (83.4 to 99.1)	315256 (298170 to 333458)	130.8 (127.7 to 134.3)	19795 (13832 to 27055)	127.8 (121.0 to 134.4)
Sierra Leone	79487 (70827 to 89089)	126.6 (119.4 to 134.3)	217837 (192320 to 261809)	189.0 (156.9 to 252.3)	14534 (10490 to 20036)	207.0 (164.6 to 288.7)
Togo	84872 (75434 to 95636)	121.4 (113.2 to 129.1)	185122 (175090 to 196437)	165.8 (161.9 to 169.7)	11574 (8013 to 16095)	162.9 (155.5 to 170.8)

Table S2: Age-standardised rates of incidence, prevalence, and years lived with disability (YLDs) of fractures by location in 2019, and percentage change from 1990 to 2019						
	Age-standardised Incidence (95% UI)		Age-standardised prevalence (95% UI)		Age-standardised YLD rate(95% UI)	
	2019 (per 100 000)	Percentage change from 1990 to 2019 (%)	2019 (per 100 000)	Percentage change from 1990 to 2019 (%)	2019 (per 100 000)	Percentage change from 1990 to 2019 (%)
Global	2296.2 (2091.1 to 2529.5)	-9.6 (-11.1 to -8.1)	5614.3 (5286.1 to 5977.5)	-6.7 (-7.6 to -5.7)	319.0 (220.1 to 442.5)	-8.4 (-9.5 to -7.2)
Central Europe, Eastern Europe, and Central Asia	4857.2 (4337.2 to 5428.1)	-17.5 (-19.1 to -15.9)	10558 (9826.7 to 11499.6)	-16.8 (-17.8 to -15.8)	571.6 (384.7 to 812.6)	-19.5 (-21.1 to -18.0)
Central Asia	3061.7 (2744.6 to 3411.4)	-8.2 (-10.4 to -5.9)	6476.9 (6073.9 to 6964.7)	-7.5 (-8.5 to -6.3)	363.8 (248 to 514.7)	-9.6 (-11.1 to -8.1)
Armenia	2913.7 (2576.1 to 3313.3)	-28.7 (-32.3 to -24.6)	6189.3 (5712.5 to 6713.5)	-33.0 (-35.6 to -30.7)	342.9 (238.4 to 479)	-36.1 (-40.0 to -33.0)
Azerbaijan	2739.6 (2428.6 to 3077.4)	-4.9 (-8.4 to -1.1)	5673.1 (5286.8 to 6119.8)	-4.6 (-6.6 to -2.2)	320.7 (217.6 to 452.8)	-7.4 (-10.2 to -4.3)
Georgia	4272.7 (3889 to 4691.5)	9.1 (5.1 to 13.6)	8551 (8001.9 to 9229.4)	8.5 (6.4 to 10.9)	477.3 (323.9 to 674.5)	7.8 (5.4 to 11.0)
Kazakhstan	3627.1 (3284.3 to 4018.7)	-4.2 (-7.6 to -.9)	7441.5 (6956.9 to 8032.5)	-4.2 (-5.6 to -2.8)	410.6 (277.3 to 582.4)	-7.2 (-9.3 to -5.2)
Kyrgyzstan	2717.6 (2423 to 3061.4)	-20.3 (-23.7 to -16.2)	5735.2 (5374.1 to 6164.2)	-19.7 (-21.2 to -18.3)	324.2 (219.6 to 458.9)	-21.6 (-23.6 to -19.7)
Mongolia	3630.2 (3284.8 to 4000.9)	16.9 (13.5 to 20.4)	7609.7 (7109.8 to 8229.4)	15.8 (13.8 to 18.0)	432.3 (292.1 to 611)	11.4 (8.5 to 14.5)
Tajikistan	2584.8 (2292.3 to 2905.4)	-16.8 (-20.0 to -13.7)	6046.5 (5548 to 6629.1)	-7.6 (-12.4 to -.2)	358.5 (252.4 to 487.6)	-6.8 (-12.7 to 3.6)
Turkmenistan	2804.4 (2509.2 to 3145.4)	5.1 (1.3 to 8.8)	5572 (5192.8 to 6026.7)	-.6 (-2.4 to 1.5)	315 (213 to 447.9)	-3.4 (-6.1 to -.7)
Uzbekistan	2911.4 (2595.1 to 3263)	-3.6 (-7.0 to -.5)	6009 (5644.1 to 6443.5)	-2.7 (-4.2 to -1.1)	337.3 (227.7 to 477.9)	-5.6 (-7.8 to -3.4)
Central Europe	5822.6 (5164.2 to 6578)	-10.0 (-12.8 to -6.7)	11608.8 (10771.5 to 12593.8)	-11.0 (-12.6 to -9.3)	627.1 (421.8 to 894.4)	-14.7 (-17.1 to -12.2)
Albania	5589.6 (4960.8 to 6317)	-9.8 (-14.8 to -4.9)	10897.4 (10087.1 to 11812.5)	-6.9 (-9.6 to -3.7)	590.6 (397.6 to 846.6)	-11.2 (-14.7 to -7.5)
Bosnia and Herzegovina	5496 (4848.3 to 6285.7)	-1.7 (-6.0 to 2.7)	11885.4 (10892.9 to 13095.8)	13.2 (8.9 to 18.9)	653.8 (453.1 to 904.1)	8.1 (3.3 to 15.1)
Bulgaria	5901.3 (5279.4 to 6637.9)	-9.9 (-13.8 to -6.3)	11482.6 (10648 to 12480.8)	-11.6 (-13.4 to -9.8)	624.2 (421 to 888.7)	-13.7 (-15.9 to -11.6)
Croatia	6164 (5506 to 6968.7)	-9.8 (-14.4 to -4.9)	12389.7 (11568.5 to 13381.7)	-8.2 (-10.5 to -5.5)	673 (456.4 to 953.7)	-9.4 (-12.2 to -6.2)
Czech Republic	6279.1 (5575.3 to 7111.9)	-12.5 (-16.4 to -8.3)	12446.6 (11580.9 to 13479.7)	-11.9 (-13.9 to -9.7)	669.2 (451.3 to 954.2)	-15.6 (-18.3 to -12.5)
Hungary	5828.6 (5099 to 6675.2)	-16.1 (-20.4 to -11.4)	11407.1 (10562.9 to 12440.5)	-17.9 (-20.4 to -15.1)	614.7 (413.1 to 880.8)	-22.2 (-25.6 to -18.7)

Montenegro	5751.2 (5091.5 to 6478)	-.1 (-3.2 to 3.1)	11409.3 (10582.6 to 12376.6)	.4 (-1.4 to 2.1)	614.6 (411.7 to 882.4)	-2.0 (-4.4 to .3)
North Macedonia	5296.5 (4683.2 to 6055.4)	-4.6 (-9.6 to .4)	10329.1 (9587.6 to 11230.6)	-3.4 (-7.2 to .2)	561 (377.5 to 802.5)	-8.0 (-12.0 to -4.1)
Poland	5777.8 (5089.6 to 6539.8)	-8.3 (-11.1 to -5.5)	11727.4 (10859.3 to 12750.5)	-10.3 (-12.1 to -8.3)	631.9 (424.9 to 900.5)	-14.5 (-17.5 to -11.7)
Romania	5584.1 (4960.1 to 6328.8)	-15.0 (-18.8 to -10.5)	10896.5 (10107.4 to 11826.4)	-17.4 (-19.2 to -15.7)	589.9 (396.4 to 845)	-20.6 (-23.0 to -18.2)
Serbia	5340.9 (4700.3 to 6119.7)	3.2 (-1.6 to 8.2)	10767.7 (9963.7 to 11675.1)	1.3 (-1.4 to 4.3)	583.3 (395.8 to 826.3)	-2.7 (-6.2 to 1.3)
Slovakia	6416.2 (5724.6 to 7210.1)	-5.2 (-8.7 to -1.2)	12595.7 (11681.3 to 13677)	-7.0 (-8.8 to -4.6)	676.9 (453.9 to 968.1)	-10.2 (-12.7 to -7.2)
Slovenia	7057.4 (6253.3 to 7989.7)	-8.8 (-12.7 to -4.7)	13743.5 (12804.8 to 14794.2)	-10.7 (-13.0 to -8.3)	744.1 (500.2 to 1055.5)	-11.7 (-14.3 to -9.1)
Eastern Europe	5222.1 (4647.7 to 5895.5)	-17.8 (-19.3 to -16.3)	11285.2 (10483.9 to 12359.7)	-17.8 (-18.8 to -16.7)	609.1 (409.4 to 866.8)	-20.2 (-21.8 to -18.7)
Belarus	5287.8 (4772.2 to 5849.1)	-4.2 (-7.8 to -.6)	10889.9 (10104.2 to 11893.6)	-7.9 (-9.8 to -5.9)	584.8 (391.2 to 833)	-11.4 (-14.0 to -8.9)
Estonia	4696.5 (4205.1 to 5243)	-28.3 (-32.0 to -24.5)	9559.2 (8875.3 to 10462.5)	-31.1 (-32.8 to -29.3)	515.6 (347.9 to 733.9)	-33.0 (-35.1 to -30.8)
Latvia	4839.2 (4378.4 to 5363.5)	-32.9 (-36.2 to -29.5)	9892.2 (9217.6 to 10727.3)	-35.6 (-36.9 to -34.2)	534.1 (359.5 to 755.3)	-37.6 (-39.4 to -35.8)
Lithuania	5170.3 (4675.4 to 5724.8)	-21.6 (-25.0 to -18.2)	10744.2 (10000.4 to 11633.6)	-24.6 (-26.0 to -22.9)	581.6 (392.4 to 825.1)	-26.3 (-28.1 to -24.3)
Moldova	4046.8 (3626.5 to 4516.4)	-23.1 (-26.4 to -19.2)	8519.2 (7943.2 to 9225.8)	-25.1 (-26.9 to -23.2)	471.3 (320.3 to 665.2)	-27.1 (-29.2 to -24.9)
Russia	5236 (4656.4 to 5925.9)	-18.8 (-20.9 to -16.7)	11475.9 (10669.1 to 12567.9)	-17.8 (-19.3 to -16.3)	618.6 (416.6 to 876.4)	-20.4 (-22.3 to -18.6)
Ukraine	5314.8 (4723.5 to 6030.4)	-15.2 (-17.3 to -13.0)	11149.3 (10290.8 to 12274)	-17.3 (-18.5 to -16.1)	603.9 (404.4 to 868.1)	-19.0 (-20.6 to -17.5)
High-income	3261.6 (2880.1 to 3712.8)	-6.8 (-8.7 to -4.8)	6767.1 (6291 to 7333.4)	-6.4 (-7.3 to -5.4)	369.2 (248.9 to 526.5)	-7.1 (-8.0 to -6.0)
Australasia	6814.8 (5934.5 to 7798.3)	2.1 (-.9 to 5.0)	12191.9 (11317.4 to 13256)	1.6 (.4 to 3.1)	662.9 (444.7 to 945.3)	1.9 (.4 to 3.6)
Australia	6652.7 (5772.2 to 7650.4)	2.4 (-1.0 to 5.8)	11911.6 (11036.1 to 12975.1)	2.4 (1.0 to 3.9)	647.6 (434.6 to 925.3)	2.6 (.9 to 4.6)
New Zealand	7698.8 (6859.8 to 8655.9)	2.0 (-2.6 to 7.0)	13728.8 (12814.8 to 14800.6)	-.4 (-3.4 to 2.2)	747 (501 to 1063.9)	-.4 (-3.6 to 2.2)
High-income Asia Pacific	3066.8 (2696 to 3501)	-7.9 (-10.3 to -5.2)	6251.8 (5813.5 to 6824)	-8.0 (-9.2 to -6.8)	345.3 (232 to 492.9)	-8.8 (-10.2 to -7.4)
Brunei	3437.8 (3078.7 to 3840.7)	-8.5 (-11.1 to -5.9)	6757.2 (6316 to 7310.8)	-12.5 (-14.1 to -11.1)	373 (252.1 to 529.5)	-14.6 (-16.9 to -12.7)
Japan	2676.1 (2334.1 to 3067.4)	-8.4 (-10.4 to -6.5)	5688.3 (5278.3 to 6230.3)	-9.4 (-10.5 to -8.3)	317.1 (213.6 to 453.7)	-9.0 (-10.2 to -7.9)

Singapore	3279.6 (2856.2 to 3779.5)	-7.3 (-10.7 to -3.3)	6408 (5965.7 to 6971.6)	-5.8 (-7.3 to -4.0)	349.8 (233.4 to 501.8)	-6.7 (-8.8 to -4.5)
South Korea	3928.1 (3474.5 to 4452.1)	-8.9 (-12.9 to -4.2)	7683.2 (7169.5 to 8335.7)	-11.2 (-13.3 to -9.0)	417.5 (280.3 to 593)	-14.8 (-17.5 to -12.2)
High-income North America	2618.6 (2330.6 to 2950.2)	-9.4 (-12.0 to -6.8)	6200.9 (5804.3 to 6647.3)	-7.2 (-8.8 to -5.6)	338.4 (230.3 to 472)	-8.0 (-9.5 to -6.3)
Canada	3224.3 (2897.9 to 3574.7)	-1.8 (-4.4 to .7)	6530.7 (6168.8 to 6965.8)	-2.0 (-3.3 to -.7)	353.7 (239.3 to 499.9)	-2.0 (-3.9 to -.1)
Greenland	3082.7 (2781 to 3416.8)	-24.6 (-26.9 to -22.1)	7231.4 (6777.6 to 7836.1)	-24.4 (-25.6 to -23.2)	409 (280.6 to 572.1)	-24.8 (-26.3 to -23.3)
USA	2550.5 (2250.8 to 2884)	-10.5 (-13.2 to -7.7)	6161.8 (5760.4 to 6613.5)	-7.9 (-9.6 to -6.1)	336.5 (229.6 to 470.1)	-8.7 (-10.3 to -6.9)
Southern Latin America	3672 (3254.1 to 4169.3)	.7 (-2.0 to 3.7)	7016.9 (6580.2 to 7558.8)	-.7 (-2.1 to .6)	387.5 (262 to 551.2)	-4.9 (-7.5 to -2.6)
Argentina	3603.8 (3199.8 to 4087.3)	-1.2 (-4.4 to 2.2)	6917.4 (6486.8 to 7469.1)	-1.9 (-3.6 to -.3)	385.1 (260.3 to 545.2)	-5.7 (-8.3 to -3.3)
Chile	3755.8 (3338.6 to 4247.3)	8.6 (5.1 to 12.2)	7120.7 (6650.2 to 7647.7)	5.7 (4.0 to 7.4)	386.4 (261.2 to 551.6)	.0 (-3.4 to 3.1)
Uruguay	4067.6 (3621.8 to 4567.4)	-11.3 (-15.8 to -6.4)	7668.9 (7191.8 to 8231.7)	-10.0 (-11.9 to -8.2)	423.2 (285.9 to 600)	-12.5 (-14.7 to -10.3)
Western Europe	3615.6 (3128.6 to 4182.8)	-5.2 (-8.3 to -1.7)	7150 (6599.8 to 7788.2)	-5.2 (-6.8 to -3.3)	387 (260.1 to 556)	-5.7 (-7.2 to -3.5)
Andorra	4083.9 (3543.7 to 4699.8)	4.9 (1.3 to 8.5)	8115.8 (7482.2 to 8796.2)	3.6 (1.7 to 5.9)	442.9 (298.2 to 635.5)	3.9 (1.7 to 6.5)
Austria	3958.4 (3448 to 4588.4)	-10.3 (-14.2 to -5.8)	7784.6 (7174.4 to 8479.7)	-9.5 (-11.5 to -6.9)	418.7 (280.4 to 599)	-9.4 (-11.7 to -6.8)
Belgium	4637 (4020 to 5370.6)	10.5 (7.2 to 14.5)	9189.7 (8450.9 to 9993.9)	10.0 (8.1 to 12.4)	494.4 (332 to 712.3)	9.8 (7.5 to 12.4)
Cyprus	3834.6 (3324.9 to 4437.6)	6.1 (2.1 to 10.3)	7363.5 (6792.9 to 8044.6)	5.0 (2.6 to 7.7)	398.7 (268.6 to 573.3)	2.4 (-.8 to 5.5)
Denmark	3681 (3140.9 to 4364)	-8.8 (-13.6 to -3.4)	7122.6 (6505.7 to 7861.2)	-8.8 (-11.6 to -5.1)	385.1 (259.7 to 552.1)	-9.7 (-12.8 to -5.9)
Finland	5306.3 (4551.2 to 6288.1)	-3.2 (-7.1 to 1.4)	10400.3 (9509.4 to 11412)	-2.8 (-5.0 to -.3)	558.1 (373.6 to 804.2)	-3.0 (-5.5 to -.3)
France	4285.4 (3731.6 to 4912.3)	-4.2 (-8.0 to -.4)	8395.1 (7764.5 to 9160.5)	-4.6 (-6.7 to -2.2)	455.7 (305.2 to 657)	-5.2 (-7.5 to -2.5)
Germany	3720.8 (3219.1 to 4327)	1.1 (-3.0 to 5.4)	7259.1 (6672.8 to 7931)	.4 (-1.6 to 2.7)	391.1 (262.8 to 562.1)	.0 (-2.3 to 2.5)
Greece	3373.3 (2909.7 to 3921.5)	-8.8 (-12.9 to -4.8)	6730.3 (6164.2 to 7381)	-9.3 (-11.4 to -6.7)	362.6 (241.6 to 526)	-9.7 (-12.1 to -6.9)
Iceland	3660.3 (3148.3 to 4284.9)	1.2 (-2.5 to 5.3)	7179.6 (6587.3 to 7894)	.5 (-1.5 to 2.7)	387.5 (260.9 to 555)	.5 (-1.8 to 2.8)
Ireland	3673.9 (3147.7 to 4303.7)	.7 (-3.6 to 5.4)	7161 (6559.7 to 7906.9)	1.9 (-.6 to 4.6)	386.2 (258.6 to 560.7)	1.0 (-1.7 to 3.7)
Israel	3282.3 (2791.1 to 3864.2)	-2.1 (-6.5 to 2.7)	6653.7 (6092.4 to 7304.3)	6.1 (3.2 to 9.8)	362.3 (247.5 to 515.1)	4.3 (1.0 to 8.7)

Italy	3089.9 (2667.9 to 3572.6)	-22.4 (-25.7 to -18.7)	6255.4 (5780.5 to 6838.2)	-21.1 (-22.8 to -19.1)	336.5 (226.6 to 485.6)	-21.5 (-23.4 to -19.3)
Luxembourg	4047.4 (3531.8 to 4652.3)	-12.0 (-16.3 to -7.9)	7946.3 (7349.9 to 8668.5)	-11.3 (-13.4 to -8.8)	428.6 (287.7 to 615.8)	-12.1 (-14.5 to -9.3)
Malta	4081.2 (3500.2 to 4790.9)	6.8 (3.1 to 10.8)	7913.8 (7221.2 to 8720.4)	8.5 (6.7 to 10.7)	426.4 (285.9 to 614.8)	7.1 (4.6 to 9.7)
Monaco	2581 (2196.6 to 3053.9)	12.4 (9.2 to 15.8)	5984.6 (5470.8 to 6572.8)	12.1 (10.4 to 13.9)	335.8 (227.6 to 480.1)	11.7 (9.3 to 14.1)
Netherlands	3402.7 (2976.8 to 3892.5)	13.2 (10.2 to 16.4)	6530.6 (6085.7 to 7046.2)	11.3 (9.7 to 13.4)	358.9 (242.8 to 511.1)	12.2 (10.0 to 14.8)
Norway	3035.9 (2628 to 3552)	-3.5 (-6.4 to -.5)	6563.4 (6024.9 to 7169.3)	-5.3 (-6.8 to -3.7)	369.4 (251.9 to 525.5)	-4.5 (-6.0 to -2.8)
Portugal	2505.6 (2208.6 to 2855.8)	-26.0 (-29.8 to -21.9)	4950.5 (4610.3 to 5352.5)	-28.1 (-30.0 to -26.4)	268.1 (180.9 to 381.7)	-29.9 (-32.1 to -27.8)
San Marino	2655.3 (2283.3 to 3135)	7.2 (3.6 to 11.1)	6098.4 (5563 to 6741.9)	7.1 (4.6 to 9.3)	342 (231.7 to 491.4)	6.8 (4.0 to 9.5)
Spain	3446.3 (2961.7 to 4048.6)	-1.8 (-8.1 to 5.6)	6874.8 (6300.6 to 7558.1)	-2.0 (-5.3 to 1.6)	370.8 (248.4 to 536.7)	-2.5 (-6.1 to 1.5)
Sweden	2812.9 (2421.3 to 3295.7)	.3 (-2.6 to 3.2)	6243.1 (5710.6 to 6834.5)	-1.6 (-3.5 to .2)	351.4 (237.9 to 501)	-1.3 (-3.3 to .9)
Switzerland	4664.2 (4035.2 to 5419.1)	-16.4 (-20.5 to -11.6)	9023.8 (8265.9 to 9905.3)	-15.9 (-18.3 to -13.3)	489 (330.1 to 710.6)	-15.7 (-18.2 to -13.0)
UK	3338.7 (2883.4 to 3870.5)	.1 (-2.9 to 3.0)	6665.8 (6133.3 to 7286)	1.2 (-.7 to 2.7)	360.8 (243.2 to 516.2)	.2 (-1.9 to 2.0)
Latin America and Caribbean	2047.6 (1841.1 to 2296)	-11.9 (-13.9 to -9.9)	4661.9 (4362.6 to 4982.5)	-12.5 (-13.5 to -11.5)	266.5 (183.2 to 371.3)	-15.7 (-17.8 to -13.9)
Andean Latin America	2002.4 (1808.2 to 2210.9)	-12.1 (-17.8 to -6.7)	4169.5 (3925.8 to 4447.5)	-1.8 (-4.4 to .4)	239.3 (163.9 to 330.3)	-9.4 (-13.9 to -5.7)
Bolivia	1861.6 (1683.9 to 2053.2)	-3.4 (-6.8 to .2)	3786.9 (3588.8 to 4061.5)	-5.6 (-7.6 to -3.5)	222.8 (153.1 to 309.9)	-9.9 (-12.8 to -7.0)
Ecuador	2206.3 (2000.2 to 2412.9)	8.4 (4.9 to 12.0)	4567.8 (4309.5 to 4896.4)	4.0 (2.0 to 6.3)	261.1 (177.7 to 364.9)	-2.8 (-6.6 to .9)
Peru	1954.8 (1760.7 to 2170.7)	-21.6 (-28.8 to -13.9)	4097.8 (3831.1 to 4389.4)	-3.6 (-7.0 to -.8)	234.1 (160.1 to 324.1)	-12.4 (-18.4 to -7.8)
Caribbean	2343.5 (2136.5 to 2564.9)	12.8 (10.3 to 15.3)	5166.9 (4867.7 to 5490.3)	15.9 (12.5 to 20.9)	301.6 (210.7 to 414.2)	15.2 (10.5 to 24.4)
Antigua and Barbuda	2418.9 (2156.4 to 2707.1)	10.1 (6.0 to 14.3)	4570 (4305.6 to 4882.9)	8.1 (6.1 to 10.2)	255.2 (173.3 to 355.5)	5.1 (2.4 to 7.8)
The Bahamas	1986.8 (1792.8 to 2195.2)	11.6 (7.9 to 15.2)	4156.4 (3942.4 to 4400.7)	6.8 (4.7 to 9.0)	236.5 (160.6 to 329.5)	4.2 (1.5 to 7.1)
Barbados	1919 (1723.9 to 2144.3)	11.6 (7.9 to 15.0)	3814.5 (3605.4 to 4041.6)	7.6 (5.9 to 9.8)	214.2 (144.7 to 298.8)	4.4 (1.6 to 7.3)
Belize	2252.1 (2041.5 to 2492.8)	10.5 (5.1 to 15.6)	4757.7 (4517.5 to 5007.2)	17.2 (15.1 to 19.6)	273.5 (187.6 to 379.1)	13.7 (10.8 to 16.6)

Bermuda	1595.4 (1402.6 to 1820.2)	14.0 (9.3 to 18.5)	3665.8 (3453.6 to 3907.3)	6.1 (3.4 to 8.8)	207.4 (140.8 to 294)	3.6 (.3 to 7.0)
Cuba	3004.8 (2722.6 to 3317.1)	21.0 (16.2 to 26.0)	5704.8 (5413.5 to 6016.6)	10.2 (7.5 to 12.5)	317.6 (216 to 445.8)	5.9 (2.6 to 9.0)
Dominica	1904.1 (1718.5 to 2104.2)	11.3 (8.0 to 14.2)	4110.1 (3892.6 to 4332.7)	13.3 (11.5 to 15.3)	235.4 (161.3 to 326.6)	11.1 (8.5 to 14.0)
Dominican Republic	2264.9 (2052.2 to 2499.5)	30.2 (26.0 to 34.4)	4891 (4660.7 to 5147.6)	26.3 (23.9 to 28.4)	280.5 (190.3 to 391.2)	20.6 (17.2 to 23.9)
Grenada	2343.7 (2117.5 to 2593.4)	22.0 (17.6 to 26.2)	4638.5 (4395.8 to 4907.1)	10.1 (3.4 to 14.9)	265.3 (181 to 368.5)	6.4 (-1.1 to 11.6)
Guyana	2459.2 (2269 to 2670.1)	12.8 (9.9 to 16.0)	5058.8 (4798.5 to 5349.5)	11.0 (9.3 to 12.8)	294.9 (204.4 to 408.9)	7.9 (5.2 to 10.4)
Haiti	1902.5 (1722.2 to 2098.3)	-2.6 (-5.3 to .5)	5390.9 (4745.4 to 6347)	31.6 (16.7 to 54.1)	348.3 (246.2 to 482.8)	39.1 (19.4 to 80.6)
Jamaica	2061.1 (1840.8 to 2311.7)	1.0 (-2.6 to 4.8)	3992.1 (3770.9 to 4253.1)	4.4 (2.7 to 6.2)	225.9 (153.1 to 315.7)	2.0 (-.6 to 4.8)
Puerto Rico	1770.4 (1575.9 to 2011.4)	7.7 (3.4 to 12.1)	4084.9 (3808.7 to 4389)	1.3 (-1.4 to 5.6)	229.7 (156.8 to 324.9)	-1.2 (-4.5 to 3.5)
Saint Kitts and Nevis	1805.3 (1604.9 to 2044.2)	9.2 (4.7 to 13.6)	3949.1 (3728.9 to 4199.7)	3.9 (2.0 to 6.0)	223.9 (152.5 to 315.3)	2.3 (-.7 to 5.1)
Saint Lucia	2074.3 (1874.1 to 2298.6)	11.6 (7.7 to 15.3)	4217.2 (3989.6 to 4463.4)	8.3 (6.5 to 10.6)	238.2 (162.3 to 332.9)	4.7 (1.9 to 7.6)
Saint Vincent and the Grenadines	2094.4 (1894.9 to 2300.2)	15.1 (11.9 to 18.4)	4231 (4008.4 to 4480.3)	14.7 (12.9 to 16.6)	242.5 (166 to 337.5)	12.1 (9.6 to 14.8)
Suriname	1945.1 (1755.6 to 2139)	14.4 (10.8 to 18.1)	4167.6 (3955.1 to 4418.2)	7.5 (2.5 to 10.3)	242.5 (165.8 to 339)	4.2 (-2.0 to 7.9)
Trinidad and Tobago	1872.8 (1689.4 to 2072.9)	1.1 (-3.0 to 5.6)	3956.8 (3746.1 to 4190.4)	7.1 (5.4 to 8.7)	223.9 (151.9 to 313)	3.1 (.2 to 6.0)
Virgin Islands	1565.4 (1391 to 1769.7)	5.5 (1.7 to 9.5)	3566.1 (3371 to 3770.1)	-1.8 (-3.7 to .4)	204 (139 to 286.1)	-4.5 (-7.1 to -1.6)
Central Latin America	2172 (1942.8 to 2434.8)	-16.7 (-19.5 to -14.1)	4840.9 (4536.3 to 5158)	-19.0 (-20.3 to -17.9)	274.9 (190 to 383.7)	-22.9 (-25.9 to -20.6)
Colombia	2305 (2050.1 to 2588.9)	-28.0 (-34.6 to -23.0)	4953.3 (4640.7 to 5274.9)	-24.4 (-26.4 to -22.4)	275.6 (189.6 to 384.4)	-28.6 (-31.5 to -25.9)
Costa Rica	2497.9 (2224.1 to 2819.3)	1.4 (-1.6 to 4.0)	4963.2 (4645.6 to 5308)	-1.2 (-2.7 to .2)	273 (184.9 to 382.9)	-5.5 (-7.9 to -3.1)
El Salvador	2424.9 (2183.3 to 2719.9)	-31.1 (-39.2 to -21.1)	6485.3 (5647.5 to 7684.2)	-24.6 (-31.1 to -17.5)	380.2 (273.4 to 514.4)	-34.8 (-45.7 to -25.1)
Guatemala	2765.2 (2499.4 to 3080.3)	-10.0 (-21.7 to .7)	6004.1 (5486.3 to 6731.9)	-9.3 (-17.6 to -2.9)	354.3 (248.9 to 484.7)	-18.6 (-31.2 to -9.3)
Honduras	2282.3 (2065.4 to 2544)	-.7 (-4.4 to 3.3)	4670.4 (4392.9 to 4969.3)	-.7 (-2.9 to 2.0)	271.7 (188.8 to 376)	-3.8 (-6.7 to -.1)
Mexico	1913.9 (1688.9 to 2186.8)	-16.9 (-18.5 to -15.1)	4491.7 (4180 to 4831.7)	-20.6 (-21.4 to -19.8)	256.4 (175.6 to 358.7)	-22.5 (-23.7 to -21.4)
Nicaragua	2033.2 (1787.4 to 2301.7)	-6.2 (-10.4 to -2.0)	5568.5 (4702.6 to 6977.8)	-26.2 (-34.0 to -18.5)	326.8 (234 to 451)	-35.3 (-47.1 to -24.9)

Panama	2219.7 (1978.9 to 2509.1)	-2.4 (-5.9 to .9)	4479.5 (4186.8 to 4824.4)	-7.7 (-10.8 to -5.7)	248.9 (168.5 to 349.1)	-11.9 (-15.9 to -9.0)
Venezuela	2657.2 (2364.1 to 2990.2)	-3.4 (-7.3 to .5)	5323.5 (4999.6 to 5683.1)	-7.3 (-8.8 to -5.6)	297.3 (204.6 to 416.4)	-11.6 (-14.3 to -9.0)
Tropical Latin America	1826.5 (1619.6 to 2094)	-11.7 (-13.4 to -10.1)	4472.4 (4142.5 to 4861.8)	-13.1 (-14.5 to -11.9)	256.1 (174.1 to 363.4)	-14.8 (-16.3 to -13.3)
Brazil	1805.3 (1599.8 to 2075.5)	-12.3 (-14.0 to -10.7)	4455.9 (4125.5 to 4845.7)	-13.5 (-14.9 to -12.2)	255.2 (173.4 to 362.2)	-15.1 (-16.6 to -13.6)
Paraguay	2378.8 (2119.6 to 2681)	-1.1 (-3.7 to 1.8)	4966.1 (4637.5 to 5322.4)	-2.2 (-3.7 to -.5)	282.6 (192.3 to 399.4)	-6.0 (-8.6 to -3.7)
North Africa and Middle East	2515 (2283.7 to 2784.4)	3.0 (-.8 to 6.8)	5538.4 (4974.4 to 6288.4)	1.8 (-4.2 to 5.8)	322.4 (228.1 to 438.2)	-3.8 (-13.3 to 2.0)
Afghanistan	4557.5 (3665.7 to 5589.4)	81.0 (58.7 to 104.4)	10973 (6795.3 to 19665.3)	-17.3 (-39.7 to 26.5)	773.1 (440.6 to 1426.7)	-27.0 (-51.9 to 26.9)
Algeria	2021.6 (1829.1 to 2248.9)	-6.3 (-9.7 to -2.9)	4544.5 (4244.7 to 4909.1)	-7.8 (-10.5 to -4.5)	255.5 (176 to 355)	-11.0 (-14.1 to -6.9)
Bahrain	2068.3 (1845.7 to 2334.1)	11.4 (7.2 to 15.6)	4297.3 (4009.6 to 4699.8)	3.6 (1.0 to 6.5)	234.5 (157.9 to 334.3)	-2.1 (-6.0 to 2.0)
Egypt	1680.8 (1505.8 to 1890.4)	11.0 (6.9 to 15.2)	3659.1 (3435.3 to 3939.5)	5.2 (2.9 to 7.4)	206.2 (140.3 to 290.9)	.3 (-3.0 to 3.7)
Iran	1978 (1766.9 to 2225.8)	-45.1 (-50.0 to -40.4)	4743.3 (4369.2 to 5182.5)	-30.3 (-34.9 to -26.3)	263 (186.4 to 360.7)	-35.5 (-41.4 to -30.6)
Iraq	2841.3 (2578 to 3159.2)	-21.7 (-26.3 to -17.4)	9199.3 (7346.2 to 11961.6)	-21.5 (-25.8 to -17.1)	553.3 (395 to 771.6)	-25.7 (-35.4 to -18.3)
Jordan	1800.7 (1595.5 to 2045.1)	.0 (-4.2 to 4.6)	3690.9 (3432.8 to 4043.9)	-4.5 (-6.7 to -2.0)	204.2 (138.6 to 291.1)	-9.4 (-12.6 to -5.9)
Kuwait	2246.7 (1988.8 to 2535.7)	-59.6 (-67.3 to -49.7)	4955 (4593.7 to 5398)	-8.1 (-11.9 to -4.7)	268.8 (182.6 to 381.6)	-16.3 (-24.1 to -10.8)
Lebanon	2048.5 (1814.1 to 2332)	-37.7 (-48.3 to -25.9)	5727.6 (4568.5 to 8092.1)	-28.9 (-40.0 to -16.1)	313.8 (215.6 to 457.5)	-38.4 (-53.1 to -23.8)
Libya	2888.4 (2593 to 3276.3)	39.0 (26.9 to 54.0)	5647.2 (5099.9 to 6348.2)	14.1 (7.6 to 23.9)	327.8 (234.8 to 445.2)	12.7 (5.3 to 24.2)
Morocco	2300.9 (2078.4 to 2545.2)	2.6 (-1.1 to 6.4)	4933.3 (4614.4 to 5349.4)	-.9 (-3.6 to 2.0)	282.5 (195.3 to 395.4)	-5.5 (-8.9 to -2.2)
Oman	2644.4 (2389.6 to 2926.4)	-.9 (-4.9 to 3.0)	5658.6 (5295.3 to 6119.9)	-6.9 (-8.8 to -4.3)	306 (207.2 to 437)	-12.0 (-15.3 to -8.8)
Palestine	2118.4 (1900.9 to 2372.9)	-35.1 (-43.6 to -25.6)	8175.3 (6325.6 to 10828.5)	16.0 (6.6 to 29.5)	492.3 (353.4 to 689.2)	10.0 (-3.2 to 28.3)
Qatar	2904.6 (2607.5 to 3257.6)	-4.4 (-8.3 to -.4)	5970.5 (5526.6 to 6567)	-10.2 (-12.5 to -8.2)	318.5 (214.9 to 459.5)	-15.5 (-18.9 to -12.5)
Saudi Arabia	5001.9 (4525.6 to 5538.9)	19.7 (14.5 to 25.1)	10661.7 (9782.8 to 11874)	18.4 (14.9 to 22.6)	572.2 (384.1 to 828.9)	9.8 (5.0 to 14.6)
Sudan	1250.6 (1125.5 to 1406.7)	-47.1 (-56.5 to -35.3)	3799.1 (3383.9 to 4427.8)	5.7 (-.3 to 14.7)	233 (165.3 to 316.8)	2.2 (-4.8 to 12.6)
Syria	3366.8 (2890 to 3957.8)	93.7 (69.6 to 120.3)	9533.9 (7144.7 to 13020.1)	134.9 (85.2 to 207.6)	561.1 (399.6 to 792.3)	129.0 (81.1 to 212.7)

Tunisia	2110.8 (1890.2 to 2369)	5.0 (1.2 to 9.1)	4378.5 (4102.6 to 4731.3)	2.0 (.0 to 4.0)	239.3 (162.2 to 341.6)	-3.2 (-6.5 to .0)
Turkey	2122.6 (1890.6 to 2386.4)	13.4 (8.4 to 18.4)	4413.4 (4114.6 to 4793.1)	15.6 (12.1 to 19.9)	243.1 (166.6 to 343.6)	7.5 (3.2 to 12.9)
United Arab Emirates	2377.3 (2157.7 to 2618.1)	-4.0 (-7.1 to -.8)	5276.2 (4954.1 to 5683.3)	-6.1 (-7.6 to -4.3)	293.4 (198.9 to 414.7)	-9.4 (-12.0 to -7.1)
Yemen	4175.4 (3398.7 to 5133)	119.6 (77.7 to 168.3)	5501.9 (4896 to 6324)	18.2 (9.6 to 30.2)	348.1 (247 to 478.7)	21.3 (11.0 to 36.6)
South Asia	2755.8 (2483.6 to 3050.7)	4.9 (2.5 to 7.2)	7093.4 (6660.1 to 7529.5)	10.8 (9.4 to 12.1)	421.2 (295 to 574.8)	7.4 (5.9 to 9.0)
Bangladesh	1738.6 (1562.4 to 1940.6)	16.4 (12.5 to 20.2)	4418.4 (4136.4 to 4703.7)	11.9 (9.2 to 14.4)	257.9 (177.1 to 358.5)	8.3 (4.9 to 11.6)
Bhutan	2417 (2197.1 to 2641.7)	22.6 (18.3 to 27.1)	5849.6 (5534.3 to 6211.4)	18.8 (16.4 to 21.3)	344.8 (238.8 to 474.4)	13.5 (10.5 to 16.9)
India	3002.3 (2701.8 to 3327.9)	2.1 (-.4 to 4.6)	7669.6 (7204.1 to 8146)	7.5 (6.1 to 8.8)	455.9 (319 to 623.1)	4.2 (2.6 to 5.7)
Nepal	2452.3 (2218.9 to 2707.5)	7.2 (4.5 to 9.7)	5902.6 (5520.2 to 6336.3)	8.3 (6.2 to 11.2)	356.3 (250.6 to 485.3)	5.8 (3.0 to 9.4)
Pakistan	1751.7 (1567.4 to 1968.7)	20.9 (18.1 to 23.7)	4712.9 (4428.9 to 5011.4)	28.4 (26.6 to 30.7)	277.1 (192.4 to 378.2)	24.4 (21.4 to 28.1)
Southeast Asia, East Asia, and Oceania	1457.3 (1320.7 to 1620.7)	15.1 (12.6 to 17.8)	3716 (3492.8 to 3946.4)	16.4 (14.6 to 18.4)	209.7 (142.5 to 296.8)	9.5 (6.4 to 12.4)
East Asia	1359.5 (1218 to 1521.2)	24.3 (20.1 to 28.8)	3608.2 (3369.4 to 3846.5)	24.6 (22.1 to 27.5)	201.6 (135.9 to 287.3)	16.9 (13.6 to 20.2)
China	1372.6 (1228 to 1537.8)	26.5 (22.1 to 31.1)	3653.5 (3408.6 to 3895.6)	26.5 (23.9 to 29.5)	204.1 (137.6 to 290.9)	18.5 (15.1 to 22.0)
North Korea	933.8 (853.4 to 1018.6)	-10.2 (-13.3 to -6.2)	2244.2 (2126.3 to 2358)	-7.2 (-9.4 to -5.1)	129.6 (87.6 to 180.1)	-8.4 (-11.0 to -5.4)
Taiwan (Province of China)	1106.3 (1014.5 to 1222.4)	-32.4 (-35.1 to -29.5)	2605.3 (2467.1 to 2754.7)	-31.6 (-33.5 to -29.8)	139.8 (93.8 to 199.4)	-34.0 (-36.3 to -31.9)
Oceania	1613 (1458.3 to 1789.2)	18.1 (14.7 to 21.4)	3735.3 (3483.6 to 3992.3)	19.7 (17.6 to 21.8)	223.9 (158.5 to 307.1)	18.4 (15.9 to 21.1)
American Samoa	1174.6 (1054.6 to 1334)	-1.7 (-4.7 to 1.9)	3079.2 (2853.1 to 3335.7)	2.6 (-.5 to 6.5)	178 (125.5 to 244.1)	1.6 (-1.9 to 6.3)
Cook Islands	1246.5 (1104.7 to 1429.5)	1.3 (-3.9 to 6.4)	3385.3 (3137.8 to 3676.2)	3.3 (1.0 to 6.3)	190.8 (131.6 to 266.6)	.8 (-2.7 to 4.3)
Micronesia (Federated States of)	1464.2 (1326.4 to 1629.7)	12.7 (9.2 to 16.3)	3371.8 (3149.9 to 3627.4)	10.4 (8.1 to 13.0)	196.3 (136.2 to 273.2)	7.2 (3.9 to 10.3)
Fiji	1291.8 (1146.7 to 1459)	7.6 (4.1 to 11.0)	2905.7 (2700.2 to 3145.6)	7.0 (4.9 to 9.0)	168.4 (116.4 to 234.7)	5.6 (2.7 to 8.4)
Guam	1146.1 (1022.5 to 1304.2)	-.4 (-4.1 to 3.3)	2969.9 (2751.2 to 3212.1)	.3 (-1.8 to 2.8)	170.7 (116.8 to 240.2)	-.4 (-3.2 to 2.7)
Kiribati	1049.2 (939.2 to 1186.5)	9.0 (5.3 to 13.0)	2474.3 (2315.6 to 2654.9)	8.9 (6.9 to 11.2)	146.9 (101.9 to 201.9)	8.5 (5.0 to 12.1)
Marshall Islands	1393.8 (1261.5 to 1553.2)	8.0 (5.0 to 11.4)	3211.5 (3013.3 to 3442.7)	7.4 (5.8 to 9.1)	188.2 (131.1 to 260.4)	5.3 (2.5 to 7.9)

Nauru	1209.3 (1090.8 to 1365.2)	11.5 (8.1 to 15.1)	3232 (3013 to 3497.1)	8.5 (6.4 to 10.5)	186.2 (127.6 to 262.6)	7.1 (3.9 to 10.1)
Niue	1180.9 (1054.2 to 1346.2)	13.2 (9.1 to 17.4)	3036.1 (2814.6 to 3286.1)	12.5 (10.0 to 15.3)	171.9 (118.6 to 240)	10.4 (7.1 to 14.7)
Northern Mariana Islands	1447.5 (1301.9 to 1630.7)	-.3 (-4.3 to 3.6)	3546.4 (3303.1 to 3852.1)	-4.8 (-7.0 to -2.1)	200.5 (136.9 to 282.9)	-5.9 (-8.7 to -2.7)
Palau	1893 (1712.3 to 2104.4)	11.4 (8.0 to 14.5)	4407.1 (4090.1 to 4795.2)	9.7 (8.1 to 11.6)	248.6 (170.9 to 347.7)	7.7 (4.7 to 10.7)
Papua New Guinea	1674.9 (1514.6 to 1854.4)	19.8 (15.8 to 23.9)	3912.5 (3656.2 to 4171.4)	22.7 (20.3 to 25.1)	237.3 (169.1 to 325.1)	21.1 (18.1 to 24.4)
Samoa	1503.9 (1355.9 to 1684)	4.6 (-.1 to 8.9)	3563.8 (3318.7 to 3830.9)	18.0 (14.8 to 22.5)	207.1 (146.2 to 284.6)	16.3 (12.5 to 22.1)
Solomon Islands	2029.3 (1812.4 to 2265.1)	14.4 (10.5 to 18.3)	4583.8 (4238.6 to 4995.3)	13.4 (11.1 to 15.7)	269.6 (187.1 to 375.5)	11.2 (8.5 to 14.2)
Tokelau	1082.6 (968.5 to 1233.3)	14.3 (10.5 to 18.0)	2772.4 (2576.7 to 3004.7)	10.8 (8.9 to 13.4)	161.8 (111.1 to 226.1)	7.8 (4.0 to 11.1)
Tonga	1294.7 (1146.8 to 1466.6)	3.0 (-1.1 to 7.2)	2883.7 (2679.8 to 3135.8)	6.2 (3.6 to 8.9)	168.5 (116.5 to 235.6)	4.1 (.7 to 7.3)
Tuvalu	1071.7 (965.4 to 1208.6)	14.4 (10.8 to 18.5)	2792.7 (2593 to 3022)	10.2 (8.1 to 12.6)	163.1 (112.3 to 229.1)	7.3 (3.8 to 11.1)
Vanuatu	1303.6 (1175.7 to 1454.9)	9.5 (6.4 to 12.7)	3226.1 (3022.6 to 3447.1)	11.9 (9.6 to 14.6)	192.5 (135.6 to 263.1)	10.9 (7.7 to 14.4)
Southeast Asia	1560.1 (1424.7 to 1719.2)	-9.0 (-11.5 to -6.5)	3852.6 (3641.5 to 4094.1)	-5.7 (-8.0 to -4.1)	223.7 (155 to 308.1)	-10.0 (-13.5 to -7.7)
Cambodia	1879.7 (1707.1 to 2064.3)	9.7 (3.5 to 15.6)	5037.4 (4328.3 to 6471.7)	-4.1 (-23.2 to 11.5)	314.1 (218.7 to 452.8)	-14.9 (-37.9 to 6.0)
Indonesia	1195.1 (1061.2 to 1347.5)	-12.0 (-13.6 to -10.2)	3314.3 (3130.2 to 3512.5)	-16.0 (-17.3 to -14.7)	195.9 (136.3 to 270.1)	-17.7 (-19.7 to -16.1)
Laos	1465.5 (1338.8 to 1603.5)	-21.7 (-35.3 to -11.1)	3446.1 (3273.9 to 3638)	-7.7 (-9.4 to -6.2)	208 (144.3 to 285.2)	-9.9 (-12.8 to -7.2)
Malaysia	1785.1 (1629.3 to 1961.6)	9.1 (5.6 to 12.7)	4137 (3929.6 to 4359.9)	5.2 (3.8 to 6.6)	230.3 (156.2 to 322.8)	-.3 (-3.6 to 2.6)
Maldives	1517.8 (1370.4 to 1691.4)	4.0 (-.2 to 8.4)	3218.2 (3033.9 to 3417.3)	-2.2 (-4.6 to .2)	177.3 (121.4 to 250.8)	-9.4 (-13.4 to -5.7)
Mauritius	1219.2 (1099.8 to 1362.2)	21.6 (17.3 to 25.9)	3146 (2960.2 to 3362.2)	17.2 (14.4 to 20.3)	177.9 (121.5 to 250.8)	14.5 (11.0 to 18.2)
Myanmar	1896.1 (1742.6 to 2071.8)	-5.4 (-8.4 to -2.6)	4462.8 (4106.5 to 4928.3)	-2.1 (-8.1 to 3.1)	272.4 (195.4 to 367.6)	-4.2 (-13.6 to 2.4)
Philippines	1564.2 (1416.9 to 1742.8)	-12.8 (-16.4 to -9.3)	3612.8 (3392.8 to 3868.7)	-3.0 (-5.9 to -1.1)	213.2 (147.3 to 291.4)	-6.3 (-10.5 to -3.6)
Seychelles	1221.7 (1105.2 to 1368.1)	1.5 (-1.9 to 5.0)	3179.3 (2994.7 to 3372.6)	3.0 (1.2 to 4.4)	182.3 (124.7 to 253.7)	-.1 (-2.6 to 2.7)
Sri Lanka	2033.9 (1860.1 to 2233.6)	-47.9 (-56.0 to -38.3)	5331.6 (4755.1 to 6070.6)	11.1 (3.9 to 21.0)	303.3 (220.5 to 410.4)	1.8 (-6.3 to 13.2)

Thailand	1780 (1625.8 to 1948.7)	-9.4 (-13.2 to -5.8)	3967.1 (3744 to 4217.1)	-12.5 (-14.5 to -10.6)	218.7 (148.4 to 309.6)	-17.9 (-21.1 to -15.0)
Timor-Leste	1408.6 (1283.8 to 1539.2)	-50.0 (-58.5 to -39.5)	5569.4 (4532.6 to 7054.4)	1.5 (-1.5 to 4.2)	367.1 (258.6 to 523.2)	-9.3 (-17.4 to -2.4)
Vietnam	1970.8 (1797.3 to 2151)	17.6 (13.5 to 21.8)	4414 (4192.7 to 4649)	17.2 (15.3 to 19.4)	250.7 (172.1 to 351.7)	9.3 (5.3 to 13.0)
Sub-Saharan Africa	1251.2 (1144 to 1376.5)	-40.8 (-47.9 to -33.0)	3781.1 (3497.3 to 4160.9)	-2.7 (-4.8 to -.1)	237.2 (169.1 to 320.5)	-5.3 (-8.0 to -2.4)
Central Sub-Saharan Africa	1303.7 (1195.4 to 1428.7)	-14.2 (-18.6 to -9.9)	3798.9 (3396 to 4367.8)	8.9 (3.4 to 16.9)	248.6 (178.8 to 336.5)	8.6 (3.0 to 17.9)
Angola	1264.1 (1147.2 to 1386.4)	-54.8 (-62.6 to -45.0)	4897.7 (4096.7 to 6107)	-11.1 (-14.0 to -8.5)	327.3 (232.4 to 461.1)	-20.5 (-29.8 to -13.5)
Central African Republic	1523.8 (1371.6 to 1706.8)	33.9 (23.1 to 46.8)	3880.1 (3438.5 to 4512.8)	39.5 (23.9 to 62.9)	268.6 (191.2 to 376.5)	56.1 (31.3 to 103.9)
Congo (Brazzaville)	1175.5 (1068.4 to 1288.5)	-4.7 (-7.3 to -2.0)	3889 (3406.9 to 4604.4)	26.6 (11.6 to 49.7)	252.8 (182.4 to 348.7)	33.9 (14.0 to 68.9)
DR Congo	1311.3 (1198.3 to 1435.9)	6.8 (3.4 to 10.4)	3458.8 (3170.9 to 3841.4)	14.4 (6.3 to 27.1)	224 (161.9 to 299.2)	20.2 (9.1 to 40.7)
Equatorial Guinea	1241.4 (1125.2 to 1364.7)	3.9 (.2 to 7.8)	2929.7 (2780.1 to 3086.8)	.5 (-1.1 to 2.0)	174.9 (121.2 to 238.4)	-3.8 (-7.2 to -.7)
Gabon	1355.4 (1232.3 to 1482.9)	-2.2 (-4.4 to .3)	3246.1 (3082 to 3408.3)	-4.6 (-5.6 to -3.7)	194.7 (136.2 to 264.9)	-6.9 (-9.1 to -4.7)
Eastern Sub-Saharan Africa	1393.9 (1271.5 to 1536.2)	-60.1 (-66.9 to -51.4)	4400.6 (3961.6 to 5083.6)	-8.5 (-11.5 to -5.2)	280.6 (200.6 to 377.6)	-12.7 (-17.1 to -8.8)
Burundi	1609.5 (1464.3 to 1772.7)	-4.3 (-7.4 to -1.2)	9565.3 (7200 to 12935.9)	125.7 (69.8 to 210.4)	687 (462.2 to 1062.5)	164.3 (86.1 to 306.2)
Comoros	1439.3 (1292.6 to 1607.2)	.2 (-2.6 to 3.5)	4434.1 (4204.8 to 4693.1)	-3.3 (-4.3 to -2.3)	272.8 (190.3 to 375.3)	-4.6 (-6.9 to -2.7)
Djibouti	1314.8 (1184.8 to 1469.6)	-29.1 (-36.9 to -20.8)	4021.3 (3769.8 to 4309.6)	.2 (-2.3 to 4.6)	246.3 (173.1 to 334.5)	-2.4 (-6.3 to 2.7)
Eritrea	1314.4 (1180.9 to 1462.8)	-89.6 (-92.3 to -84.8)	7941.7 (5864.5 to 11722.4)	-35.7 (-40.9 to -30.2)	538.2 (357.6 to 824.9)	-47.3 (-56.6 to -38.1)
Ethiopia	1523.6 (1381.4 to 1682.1)	-81.1 (-85.4 to -75.0)	4453.2 (3947.4 to 5439.6)	-25.7 (-31.6 to -15.1)	288.2 (203.2 to 402.8)	-30.9 (-39.2 to -19.3)
Kenya	1549.2 (1401.8 to 1706.2)	1.5 (.6 to 2.4)	3668.6 (3468.9 to 3865.9)	2.4 (1.3 to 3.9)	224.8 (157.8 to 304.9)	1.6 (.2 to 3.7)
Madagascar	1245.2 (1118.8 to 1396.4)	-3.2 (-6.5 to .1)	3623.1 (3403 to 3850.3)	-7.9 (-9.3 to -6.6)	222.9 (154.1 to 308)	-8.1 (-10.4 to -5.9)
Malawi	1113.8 (997.6 to 1253.1)	2.5 (-.2 to 5.4)	3155.1 (2967.9 to 3356.5)	-2.4 (-3.8 to -1.0)	191.8 (133.5 to 265.2)	-2.6 (-5.0 to -.4)
Mozambique	1594.3 (1435.7 to 1780.3)	-21.4 (-36.9 to -5.1)	5691.1 (5036.3 to 6807.8)	-10.9 (-23.4 to -.7)	354.4 (249.6 to 485.3)	-22.3 (-40.0 to -7.7)
Rwanda	1285.5 (1153.9 to 1440.8)	-59.0 (-66.5 to -48.8)	6929.9 (5432.5 to 9245)	35.7 (6.9 to 80.6)	453 (306.2 to 687.7)	39.5 (6.4 to 99.9)

Somalia	1615.9 (1426.3 to 1844.3)	-45.6 (-55.0 to -33.9)	4459.6 (3866.8 to 5630.3)	6.8 (-10.4 to 21.9)	295.9 (208.2 to 422.9)	6.1 (-18.4 to 26.6)
South Sudan	1525.4 (1369.4 to 1717.1)	12.8 (6.0 to 20.7)	5054.9 (4423.5 to 6147.1)	8.7 (1.3 to 20.3)	335.3 (241 to 466.4)	12.6 (3.4 to 26.4)
Tanzania	1185.1 (1063.7 to 1332.6)	-.1 (-2.8 to 2.6)	3414.3 (3209.8 to 3616.2)	-1.9 (-3.0 to -.8)	207.8 (144.2 to 284.8)	-2.0 (-4.2 to .1)
Uganda	1192.5 (1069.2 to 1340.5)	-13.1 (-18.7 to -7.5)	4284.4 (3779.9 to 5151.9)	-14.6 (-23.6 to -7.4)	270 (191.9 to 366.6)	-20.0 (-33.3 to -9.5)
Zambia	1156.4 (1040.2 to 1290.8)	1.2 (-2.3 to 4.4)	3201.2 (3005.1 to 3390.3)	-1.1 (-2.3 to .3)	191.9 (132.5 to 264.1)	-2.9 (-5.3 to -.7)
Southern Sub-Saharan Africa	1056.1 (952.9 to 1182.2)	-15.8 (-17.9 to -13.7)	2838.2 (2667.1 to 3024.1)	-17.1 (-18.6 to -16.0)	168.8 (116.8 to 231.4)	-19.4 (-21.4 to -17.8)
Botswana	1128.3 (1020.9 to 1252.3)	7.4 (4.3 to 11.0)	2975.9 (2810 to 3159.9)	7.2 (5.9 to 8.9)	173.5 (119.2 to 239.9)	3.7 (1.1 to 6.4)
Eswatini	1078.5 (973.3 to 1196.8)	9.8 (6.8 to 12.8)	2921.2 (2750.8 to 3107)	10.0 (8.5 to 11.5)	172.5 (119.8 to 237.6)	5.8 (3.0 to 8.6)
Lesotho	1153.9 (1047.6 to 1272.8)	17.9 (14.5 to 21.4)	3118.2 (2935.6 to 3317.3)	25.5 (23.7 to 27.2)	185.5 (128.8 to 254.3)	21.0 (17.6 to 24.3)
Namibia	1111.8 (1003.6 to 1236.5)	3.9 (1.1 to 6.7)	3417.6 (3140.3 to 3801.5)	-11.1 (-19.0 to -5.6)	206.6 (147.4 to 276.1)	-18.9 (-30.2 to -10.6)
South Africa	1064.2 (955.6 to 1199.2)	-21.9 (-24.3 to -19.5)	2871.7 (2690 to 3062.4)	-21.8 (-23.5 to -20.5)	170.1 (117.2 to 233.6)	-24.1 (-26.2 to -22.5)
Zimbabwe	999.9 (908.7 to 1103.8)	5.5 (1.8 to 10.2)	2468.2 (2333.5 to 2611.4)	-3.3 (-4.6 to -2.0)	150.5 (104 to 205.5)	-3.0 (-5.4 to -.5)
Western Sub-Saharan Africa	1153.1 (1047.2 to 1268.9)	-1.9 (-3.9 to .1)	3475.9 (3288.5 to 3689.3)	6.4 (4.8 to 9.1)	213.5 (149.9 to 287)	5.6 (3.7 to 9.0)
Benin	1111.9 (1002.7 to 1233.7)	2.9 (.2 to 5.9)	3445.1 (3273.8 to 3631)	1.6 (.3 to 2.8)	212.2 (148.7 to 290.9)	.9 (-1.5 to 3.3)
Burkina Faso	1335.7 (1212.1 to 1479.4)	30.0 (23.1 to 36.8)	3663.1 (3470 to 3878.8)	13.2 (12.2 to 14.2)	227.5 (158.7 to 310.8)	13.4 (11.1 to 15.8)
Cameroon	1338.6 (1209.6 to 1481.6)	14.8 (10.4 to 19.6)	4037.3 (3830.6 to 4265.8)	4.6 (3.4 to 6.2)	245.5 (170.2 to 333.4)	2.9 (.1 to 5.4)
Cape Verde	1153.9 (1044.9 to 1276.2)	17.0 (14.0 to 20.1)	3111.8 (2944.5 to 3300.1)	13.4 (12.0 to 14.8)	183.6 (125.9 to 252.6)	8.2 (5.1 to 11.2)
Chad	1167.4 (1060.3 to 1285.6)	-22.0 (-30.1 to -13.0)	4006 (3665.1 to 4533.2)	7.4 (2.9 to 10.5)	256.3 (183 to 343.3)	1.6 (-6.5 to 7.7)
Cote d'Ivoire	1148.3 (1035.4 to 1267.2)	-.3 (-2.6 to 2.2)	3568.1 (3381.8 to 3779.8)	.1 (-1.1 to 1.6)	219 (154.7 to 297.4)	1.1 (-1.4 to 3.7)
The Gambia	1039.7 (937.3 to 1147.2)	5.7 (3.4 to 8.2)	3218.4 (3047.1 to 3407)	1.3 (-1.8 to 3.3)	198 (140.4 to 267.2)	-1.3 (-6.3 to 2.4)
Ghana	1264 (1133.2 to 1408.2)	15.7 (12.9 to 19.2)	3980.1 (3747 to 4247.6)	13.6 (12.6 to 14.5)	240.7 (167 to 330.9)	11.4 (9.0 to 13.6)
Guinea	1129.2 (1022 to 1250.7)	9.0 (6.0 to 12.2)	3628.7 (3435.6 to 3849.3)	11.6 (10.2 to 13.2)	224.8 (158.2 to 301.9)	10.0 (7.4 to 12.9)

Guinea-Bissau	1208.4 (1084.2 to 1344.7)	-1.6 (-4.3 to 1.0)	4090.1 (3844 to 4377.3)	-.6 (-2.4 to 2.7)	253.8 (179.6 to 343.9)	-.2 (-3.1 to 4.0)
Liberia	955.2 (860.2 to 1057.8)	-89.1 (-91.8 to -84.4)	4172.6 (3531.2 to 5376.6)	-3.6 (-17.5 to 15.0)	272.8 (194.9 to 381.4)	-13.1 (-30.6 to 8.8)
Mali	1272.3 (1148.5 to 1416.8)	-6.7 (-10.0 to -3.4)	3719.8 (3421.4 to 4150.4)	13.4 (6.4 to 26.7)	238.6 (170.3 to 317.6)	15.6 (6.4 to 31.9)
Mauritania	1185.8 (1065.5 to 1326.8)	-11.8 (-14.9 to -8.5)	3779.1 (3567.3 to 4022.4)	-12.7 (-13.7 to -11.7)	230.7 (160.4 to 315.4)	-14.7 (-16.6 to -12.7)
Niger	1137.7 (1034.2 to 1249.2)	-2.7 (-4.9 to -.2)	3246 (3079.5 to 3433.5)	-.5 (-1.5 to .7)	203.2 (143.1 to 275)	-1.3 (-3.6 to 1.1)
Nigeria	1100.5 (993.3 to 1212.8)	5.6 (3.0 to 8.4)	3190.9 (3019.6 to 3372.8)	4.1 (2.4 to 7.0)	193.7 (135.3 to 262.1)	3.7 (1.4 to 7.7)
São Tomé and PrÍncipe	1344 (1218.7 to 1480.5)	15.7 (12.6 to 18.6)	3842.9 (3635 to 4072.8)	14.9 (13.3 to 16.5)	229.9 (158.6 to 315.8)	11.4 (8.7 to 14.2)
Senegal	1051.4 (949 to 1166.4)	-1.8 (-5.0 to 1.3)	3277.3 (3106.7 to 3468.6)	3.4 (2.1 to 4.8)	202.7 (142.6 to 273.6)	2.4 (.1 to 4.8)
Sierra Leone	1055.7 (952 to 1172)	4.6 (2.0 to 7.5)	4232.1 (3780.2 to 5036)	32.8 (19.7 to 58.7)	274 (197.7 to 369.7)	38.8 (21.9 to 71.3)
Togo	1144.2 (1029.3 to 1272.8)	2.2 (-.6 to 4.7)	3570.9 (3384.7 to 3789.8)	.1 (-.9 to 1.2)	219.2 (153.1 to 301.4)	-.5 (-2.7 to 1.7)

YLD = years lived with disability

Table S3: short- and long-term disability weights for each of 12 fracture sites, for all locations and years

Fracture site	Short-term disability weight	Long-term disability weight
Fracture of clavicle, scapula, or humerus	0.0349	0.0349
Fracture of face bones	0.0669	0.0669
Fracture of foot bones except ankle	0.0260	0.0264
Fracture of hand (wrist and other distal part of hand)	0.0099	0.0138
Fracture of hip	0.2575	0.1551
Fracture of patella, tibia or fibula, or ankle	0.0501	0.0554
Fracture of pelvis	0.2788	0.1823
Fracture of radius and/or ulna	0.0281	0.0435
Fracture of skull	0.0714	0.0714
Fracture of sternum and/or fracture of one or more ribs	0.1027	0.1027
Fracture of vertebral column	0.1106	0.1106
Fracture of femur, other than femoral neck	0.1114	0.0420

Author Contributions

Managing the estimation or publications process

Ai-Min Wu, Catherine Bisignano, Theo Vos

Writing the first draft of the manuscript

Ai-Min Wu, Catherine Bisignano, Spencer L James, Theo Vos

Primary responsibility for applying analytical methods to produce estimates

Ai-Min Wu, Spencer L James, Theo Vos

Primary responsibility for seeking, cataloguing, extracting, or cleaning data; designing or coding figures and tables

Ai-Min Wu, Theo Vos

Providing data or critical feedback on data sources

Gdiom Gebreheat Abady, Jalal Arabloo, Wondwossen Niguse Asmare, Maciej Banach, Tesega Tesega Mengistu Birhanu, Srinivasa Rao Bolla, Luis Alberto Cámera, Daniel Youngwhan Cho, Rosa A S Couto, Lalit Dandona, Rakhi Dandona, Farshad Farzadfar, Irina Filip, Juanita A Haagsma, Arvin Haj-Mirzaian, Mikk Jürisson, Taras Kavetskyy, Rovshan Khalilov, Maseer Khan, G Anil Kumar, Savita Lasrado, Stephen S Lim, Zichen Liu, Ali Manafi, Navid Manafi, Ritesh G Menezes, Bartosz Miazgowski, Ted R Miller, Abdollah Mohammadian-Hafshejani, Ali H Mokdad, Christopher J L Murray, Cuong Tat Nguyen, Andrew T Olagunju, Jagadish Rao Padubidri, Jeevan Pereira, Hai Quang Pham, Marina Pinheiro, Suzanne Polinder, Navid Rabiee, Amir Radfar, Mohammad Hifz Ur Rahman, David Laith Rawaf, Salman Rawaf, Mohammad Reza Saeb, Abdallah M Samy, Lidia Sanchez Riera, David C Schwebel, Saeed Shahabi, Masood Ali Shaikh, Amin Soheili, Rafael Tabarés-Seisdedos, Marcos Roberto Tovani-Palone, Bach Xuan Tran, Ravensara S Travillian, Pascual R Valdez, Tommi Juhani Vasankari, Narayanaswamy Venketasubramanian, Giang Thu Vu, and Theo Vos.

Development of methods or computational machinery

Spencer L James, Xiaochen Dai, Juanita A Haagsma, Taras Kavetskyy, Rovshan Khalilov, Stephen S Lim, Zichen Liu, Ali H Mokdad, Christopher J L Murray, Abdallah M Samy, and Theo Vos.

Providing critical feedback on methods or results

Ai-Min Wu, Spencer L James, Gdiom Gebreheat Abady, Aidin Abedi, Eman Abu-Gharbieh, Robert Kaba Alhassan, Vahid Alipour, Jalal Arabloo, Wondwossen Niguse Asmare, Atalel Fentahun Awedew, Maciej Banach, Srikanta K Banerjee, Ali Bijani, Tesega Tesega Mengistu Birhanu, Srinivasa Rao Bolla, Luis Alberto Cámera, Jung-Chen Chang, Daniel Youngwhan Cho, Michael T Chung, Xiaochen Dai, Lalit Dandona, Rakhi Dandona, Farshad Farzadfar, Irina Filip, Florian Fischer, Artem Alekseevich Fomenkov, Tiffany K Gill, Bhawna Gupta, Juanita A

Haagsma, Samer Hamidi, Simon I Hay, Irena M Ilic, Milena D Ilic, Rebecca Q Ivers, Mikk Jürisson, Rohollah Kalhor, Tanuj Kanchan, Taras Kavetskyy, Rovshan Khalilov, Ejaz Ahmad Khan, Maseer Khan, Cameron J Kneib, Vijay Krishnamoorthy, G Anil Kumar, Narinder Kumar, Savita Lasrado, Stephen S Lim, Ali Manafi, Navid Manafi, Ritesh G Menezes, Tuomo J Meretoja, Ted R Miller, Yousef Mohammad, Abdollah Mohammadian-Hafshejani, Ali H Mokdad, Christopher J L Murray, Mehdi Naderi, Mukhammad David Naimzada, Vinod C Nayak, Cuong Tat Nguyen, Rajan Nikbakhsh, Andrew T Olagunju, Nikita Otstavnov, Stanislav S Otstavnov, Jagadish Rao Padubidri, Hai Quang Pham, Hadis Pourchamani, Navid Rabiee, Amir Radfar, Mohammad Hifz Ur Rahman, David Laith Rawaf, Salman Rawaf, Mohammad Reza Saeb, Abdallah M Samy, Lidia Sanchez Riera, David C Schwebel, Saeed Shahabi, Masood Ali Shaikh, Amin Soheili, Rafael Tabarés-Seisdedos, Marcos Roberto Tovani-Palone, Bach Xuan Tran, Ravensara S Travillian, Pascual R Valdez, Diana Zuleika Velazquez, Narayanaswamy Venketasubramanian, Giang Thu Vu, Zhi-Jiang Zhang, and Theo Vos.

Drafting the manuscript or revising it critically for important intellectual content

Ai-Min Wu, Catherine Bisignano, Spencer L James, Gdiom Gebreheat Abady, Aidin Abedi, Eman Abu-Gharbieh, Robert Kaba Alhassan, Jalal Arabloo, Malke Asaad, Atalel Fentahun Awedew, Maciej Banach, Srikanta K Banerjee, Srinivasa Rao Bolla, Daniel Youngwhan Cho, Irina Filip, Florian Fischer, Tiffany K Gill, Juanita A Haagsma, Simon I Hay, Irena M Ilic, Milena D Ilic, Rebecca Q Ivers, Mikk Jürisson, Ejaz Ahmad Khan, Maseer Khan, Ratilal Lalloo, Savita Lasrado, Ali Manafi, Navid Manafi, Ritesh G Menezes, Tuomo J Meretoja, Ted R Miller, Yousef Mohammad, Abdollah Mohammadian-Hafshejani, Mehdi Naderi, Mukhammad David Naimzada, Vinod C Nayak, Cuong Tat Nguyen, Rajan Nikbakhsh, Andrew T Olagunju, Nikita Otstavnov, Stanislav S Otstavnov, Jagadish Rao Padubidri, Hai Quang Pham, Suzanne Polinder, Hadis Pourchamani, Amir Radfar, Mohammad Hifz Ur Rahman, David Laith Rawaf, Salman Rawaf, David C Schwebel, Saeed Shahabi, Amin Soheili, Rafael Tabarés-Seisdedos, Marcos Roberto Tovani-Palone, Bach Xuan Tran, Ravensara S Travillian, Tommi Juhani Vasankari, Diana Zuleika Velazquez, Narayanaswamy Venketasubramanian, Giang Thu Vu, and Theo Vos.

Managing the overall research enterprise

Lalit Dandona, Simon I Hay, Stephen S Lim, Ali H Mokdad, Christopher J L Murray, and Theo Vos.

Members of the core research team for this topic area had full access to the underlying data used to generate estimates presented in this paper (including Lalit Dandona, Simon I Hay, Stephen S Lim, Ali H Mokdad, Christopher J L Murray, and Theo Vos). All other authors had access to, and reviewed, estimates as part of the research evaluation process, which includes additional stages of formal review.
